# Neural Algorithm Aided Operation of CO_2_ Electrolyzers

**DOI:** 10.1021/acsenergylett.5c01133

**Published:** 2025-07-17

**Authors:** Angelika A. Samu, Dániel Horváth, Balázs Endrődi, László Vidács, Csaba Janáky

**Affiliations:** † Department of Physical Chemistry and Materials Science, 37442 University of Szeged, Aradi sq. 1, Szeged 6720, Hungary; ‡ eChemicles Zrt, Alsó Kikötő sor 11, Szeged 6726, Hungary; § Department of Software Engineering, University of Szeged, Szeged, Árpád sq. 2. Szeged 6720, Hungary

## Abstract

While the number of reports on the electrochemical carbon dioxide reduction increases at an ever-accelerating rate, achieving long-term stable, selective, and energy efficient operation is still challenging. This can be attributed mostly to the short length of lab-scale measurements and the complexity of cell operation parameters. Here we introduce a high-throughput cell operation testing methodology, including data evaluation and process optimization by machine learning algorithms. An autonomously operating test station allowed collection of enough data to develop an artificial neural network model. When the model is trained on a fraction of a large data set, predictions for the operation of the same cell under different conditions are very precise. Accurate predictions can also be made for newly assembled cells and at parameter settings outside of the training parameter space. Our results pave the way for the long-term stable operation of CO_2_ electrolyzers by the adaptive optimization of the process conditions based on machine-learning-based holistic data evaluation.

## Graphical abstract

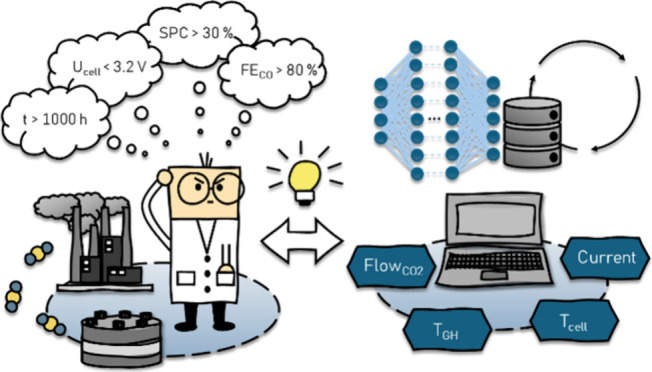

The opportunity of simultaneously reducing the emission of a harmful greenhouse gas and forming valuable chemicals attracted many researchers to focus on the electrochemical reduction of CO_2_ (CO2RR).^1–3^ This resulted in the rapid advancement of the field, considering all important process metrics (reaction rate, selectivity, stability, energy efficiency).^4,5^ This progress was facilitated by the improvement of the employed systems, cell architectures, catalysts, and other structural elements.^6–8^ Still, achieving the optimal combination of these key performance indicators (KPIs) is a grand challenge to be solved, so that CO2RR can mature into an industrially applied technology at scale.^3,9,10^

Despite the continuous gathering of experimental data (at a still accelerating rate), modeling cell operation is still in its infancy with scarce reports.^11–13^ In addition, there is still no general model that would fully describe the operation of CO2RR cells and aid in optimizing their operation on a continuous parameter space. This is not surprising, considering the large number of operational parameters,^14^ including the cell temperature, gas humidification, gas flow rate, anolyte composition, and the cell voltage/current.^15–19^ In addition, there is no standard test protocol, which also means that different research groups measure data under very different circumstances, which are hard to compare. Aging is another factor to be considered: if a cell was operated under conditions that cause (ir)­reversible changes, the subsequently recorded results might be misleading. Typical examples are electrode flooding and salt precipitation, which both alter the cell chemistry.^20,21^ Furthermore, a transient behavior might be captured over very short measurements, hence, the conclusions derived can be less relevant for continuously operating electrolyzer cells.

Advanced computational methods offer a solution to circumvent such difficulties by evaluating a large amount of experimental data together, as a whole. Machine learning (ML) is getting widely applied for predicting and optimizing the operation of water electrolyzer cells and fuel-cells, which could serve as a guide for the field of CO_2_ electrolysis and other power-to-X technologies.^22,23^ Such methods are already in use in some domains; such as identifying catalysts and additives for CO2RR.^24,25^

Despite some promising examples,^11–13^ formulating exact theoretical equations for such a complex system as CO2RR is often challenging, but this challenge can be tackled using ML.^26^ Artificial neural network (ANN) is a particularly useful ML tool to uncover complex and nonlinear relationships among variables in vast data sets. These methods rely on models that are built upon a large amount of experimental data and are validated under randomly chosen, previously unseen conditions. In addition, such data analysis can be used (i) to diagnose cell status, (ii) to ensure continuous operation, with parameters adjusted to the incidentally changing cell properties (i.e., aging), and (iii) to forecast (and possibly avoid) cell failure. These use cases have been investigated regarding hydrogen production, but are yet to be explored for CO2RR.^27^

Here we report on the collection of a large amount of reliable experimental data using our custom-built and autonomously operated electrolyzer test station. The task-tailored software allowed for filtering data that was collected during parameter settling, hence providing reproducibility. Filtering on the test station is a relatively straightforward process, as it only considers a small variance that is allowed during the measurement process. Data points that fall outside these predefined intervals (e.g., cell temperature deviates by more than 2 °C from the target setting), will not be considered for future learning processes. In this context, a data point (or measurement point) means a single row of collected values recorded in a single point of time (e.g., current, voltage, product composition, and flow rate).

The large amount of data was processed using ML algorithms to develop a model for the continuous description of cell operation in the entire parameter space, instead of the scattered experimental points. Furthermore, the ANN-based evaluation made it possible to identify overly narrowed parameter limits for optimal cell operation (e.g., too high minimal gas humidification temperature), as the model points beyond the parameter space that we originally defined based on our former experience and state-of-the-art results. ANN-based optimization of cell operation does not require exact knowledge of all physical/chemical phenomena occurring in the electrolyzer cell, nor does it require formulating exact equations. Accounting for cell aging is also possible via ANN-based methods, even without identifying the cause of performance fading. Implementing ANN-based operation in process control systems for continuous, long-term operation of CO_2_ electrolyzer is therefore one of the most realistic routes for the industrialization of CO2RR. We note that this is not in contrast with physics-based semiempirical description of cell operation.^11–13^ On the contrary, the parallel development of these methods and their final merging can provide a full description.

Our workflow consists of three key steps (Figure 1). First, raw data collected from the electrochemical measurements were preprocessed to remove outliers and faulty samples (e.g., parameter combinations that did not reach steady state conditions within the specified time limit), since these can deteriorate model prediction performance. When applying outlier detection, we only consider our 5 target variables (cell voltage, CO_2_ concentration, CO concentration, H_2_ concentration, and cathode gas flow rate). Outlier detection is performed via calculating the Z-scores of every individual output value, defined by the following equation.

$$Z=\frac{x-\mu }{\sigma }$$

where *x* is the measured value of an individual data point, μ is the mean, and σ is the standard deviation value of the distribution that the specific *x* value came from, which is defined by the entire measurement sequence of a single parameter setting. We defined a Z-score tolerance of 3. If even one of the measured values falls outside this interval, then it will be excluded. The data was split into train, validation, and test sets. In the next step, an ANN model was trained to predict cell voltage, gas composition (V/V% of CO, CO_2_, and H_2_), and output flow rate (flow out) using the input parameters: current, gas humidifier (GH) temperature (determining the humidity level of the CO_2_ inlet feed), anolyte’s electric conductivity (EC), cell temperature, and CO_2_ inlet flow rate. In the last step, the trained ANN was combined with a genetic algorithm to explore an extended parameter space and find the best parameter combinations for the optimal operation of the electrochemical cell. This last step was only achieved in part in this study. The reason behind this is the experienced cell-to-cell variation, as described below.

**Figure 1:**
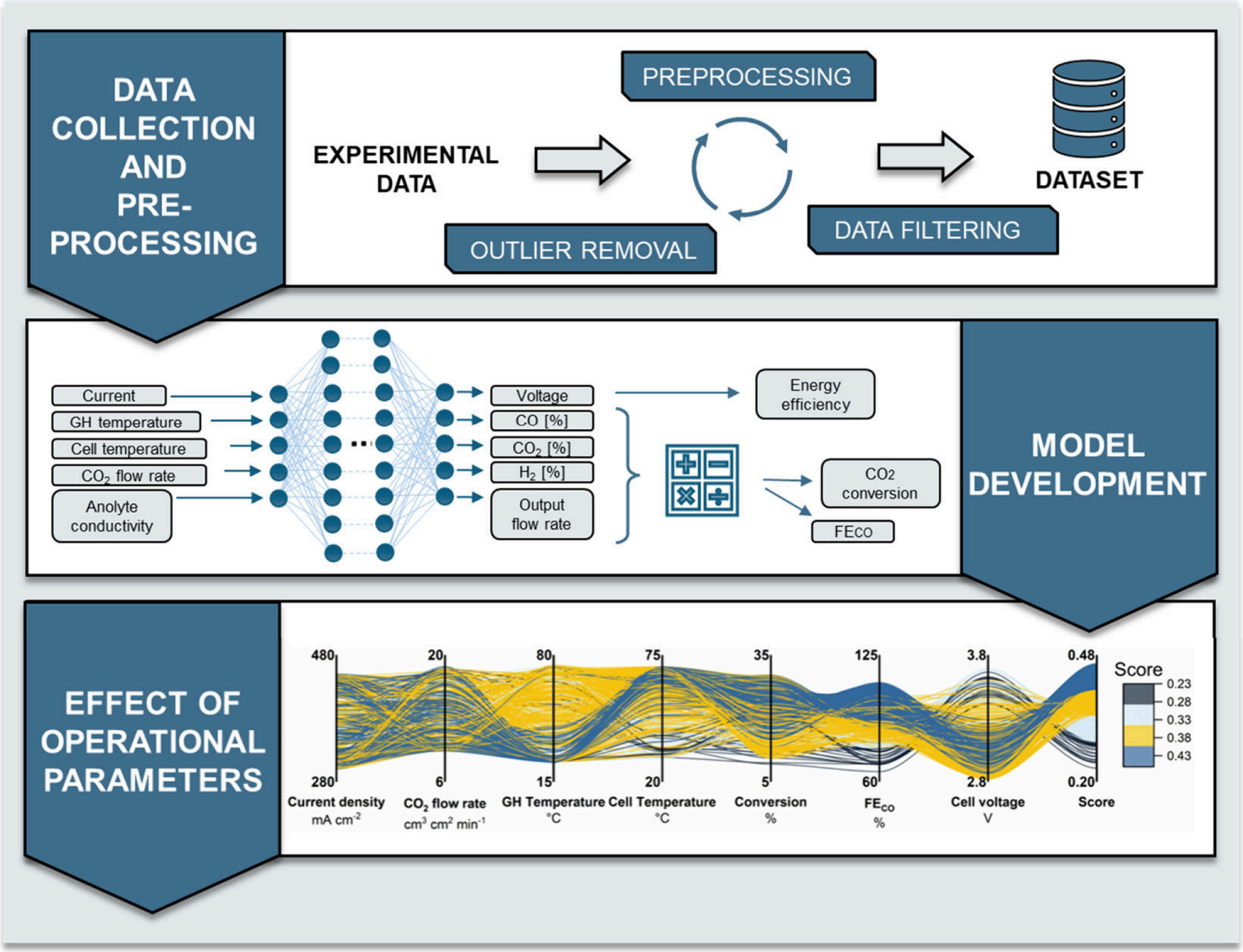
Workflow for finding the optimal parameter values for the operation of the CO_2_ electrolyzer cell.

Experimental data was collected for model training by varying 4 process parameters (current density, cell temperature, GH temperature, and CO_2_ inlet flow-rate) systematically, performing experiments at all possible combinations (see Table 1), adding up to 108 different parameter combinations, each resulting in approximately 240 individual measurement data points (1200 s sampling with 5 s interval), forming a set of ∼ 25,920 individual observations per data set. (Figures S1 and S2). In the model development phase, we evaluated multiple model architectures (see the Supporting Information (SI), particularly Table S3 and Figure S3), ultimately selecting a fully connected neural network to capture the relationship between the input and target variables. The model consists of three key parts: the input layer, the hidden layers, and the output layer. The input layer has a neuron number equal to the number of input variables, while the output layer maps weights from the hidden layers to target variables. In this case, both the number of input- and target variables were 5. Between the input- and output layers there are 3 hidden layers with 132 neurons each. To handle nonlinear relationships in the data, the network implements GELU activation function between each layer.^26^ The number of hidden layers and the number of neurons were selected based on multiple hyper-parameter optimization runs. The parameters with the best results have been selected to serve as the model hyper-parameters for the experiments. To train the model, we used *Mean Squared Error* (MSE) as the loss function and chose *AdamW* as the optimization algorithm.^28^

**Table 1:** Parameter Values Used for Model Training Electrolysis Experiments

current density/mA cm^–2^	300	350	400	
cell temperature/°C	60	65	70	
GH temperature/°C	55	60	65	
CO_2_ feed flow rate/cm^3^ min^–1^ cm^–2^	9.375	10.375	11.5	12.5

In the data preprocessing step, the test split was carefully separated from the rest of the data, avoiding any overlap between the training and test data sets. This means that parameter combinations used for testing are in no way part of the training or the validation sets, ensuring that the model testing gives a fair picture of the model’s performance. The test data set was 20% of the total unique parameter combinations (i.e., 21 randomly chosen parameter combinations out of the total 108, containing all individual observations to that particular parameter setting). The remaining 80% of parameter combinations were further split into training (80%) and validation data sets (20%).

As shown in Figure 2, Table S3 and Figure S4, the values predicted from the model match very closely with the measured results (R^2^ values above 0.98 were reached for all target values predicted by the model). This model (trained on a data set from repeating the 108 measurements on the same cell twice in a row) was used by the NSGA-II algorithm to identify favorable parameter combinations in an almost continuous parameter space (see Figure S5 and the corresponding description).^29^ The budget for the optimization was defined in terms of the maximum number of jobs (i.e., the number of parameter combinations that the algorithm is allowed to test before termination). We set this budget to 10,000. The parallel coordinates plot shown in Figure 1, where only results with 1000 test runs are shown for better visibility, represent these different scenarios, where the cell operation is predicted by the model for each connected input parameter combination. The last three axes before the score column represent the predicted values of the different target variables, while the score metric itself signifies the overall goodness of the given parameter combination. The score metric is not a target variable; it is simply calculated by taking the mean value of the individual scores of the three target variables.

**Figure 2:**
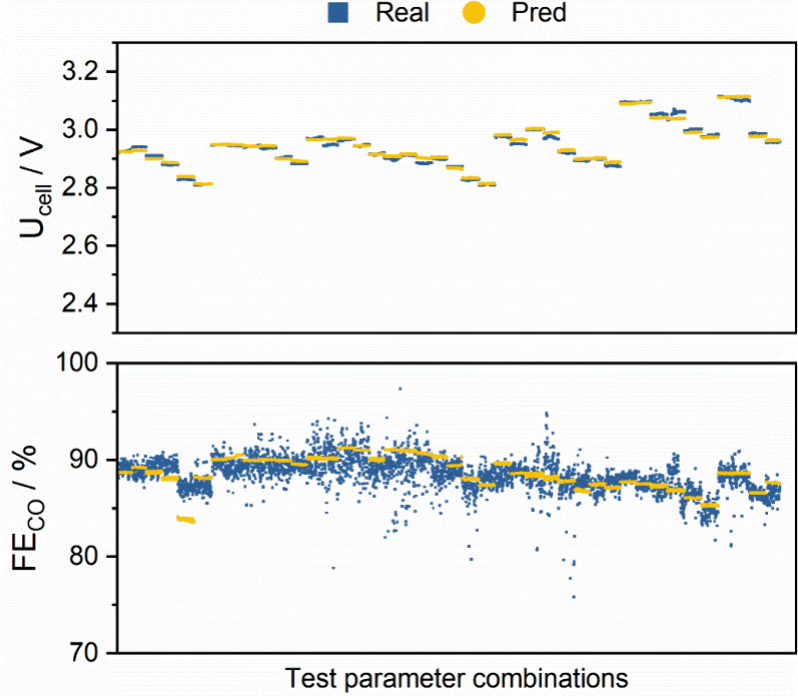
Illustration of the validity of the developed model on the test set. The model was trained via the measurement built from varying experimental parameters according to Table 1 and Figure S1. The test set was 20% of the total number of parameter combinations, carefully separated from the rest without any overlap. The order of the shown parameter combinations is entirely arbitrary, as these parameter combinations were chosen randomly and were not ordered by timestamp after selection. However, the data points inside a single measurement sequence (given parameter combination) are ordered by their timestamp.

Furthermore, 8 more parameter combinations were chosen based on the parameter optimization experiment to investigate the validity of the model, and whether the parameter space is worth extending in any direction. We refer to these parameter combinations as the *external test set*. Seven out of the 8 combinations contained at least one parameter that had a value outside of the originally defined parameter space. After a short (1 h long) conditioning step at conditions allowing relatively stable cell operation,^9^ the measurements generated from these parameter combinations was performed with a newly assembled (but otherwise identical) CO_2_ electrolyzer cell, twice in a row (without any stops, see Figure 3 and Figure 4). Note that although only 16 parameter combinations were defined (8 settings, repeated twice in a row), and results are only collected for 20 min at each setting, the duration of this whole experiment was almost 24 h. This is an important attribute of our experimental setup and control software: all important process parameters are continuously monitored, and data is only collected (for evaluation) when the target values are reached and stabilized. This is very important, especially in the case of changing the temperature of the electrolyzer cell and/or the GH, devices with rather large heat capacitance. In other cases, for example, when only the applied current density is changed, reaching the desired setting is almost instantaneous.

**Figure 3:**
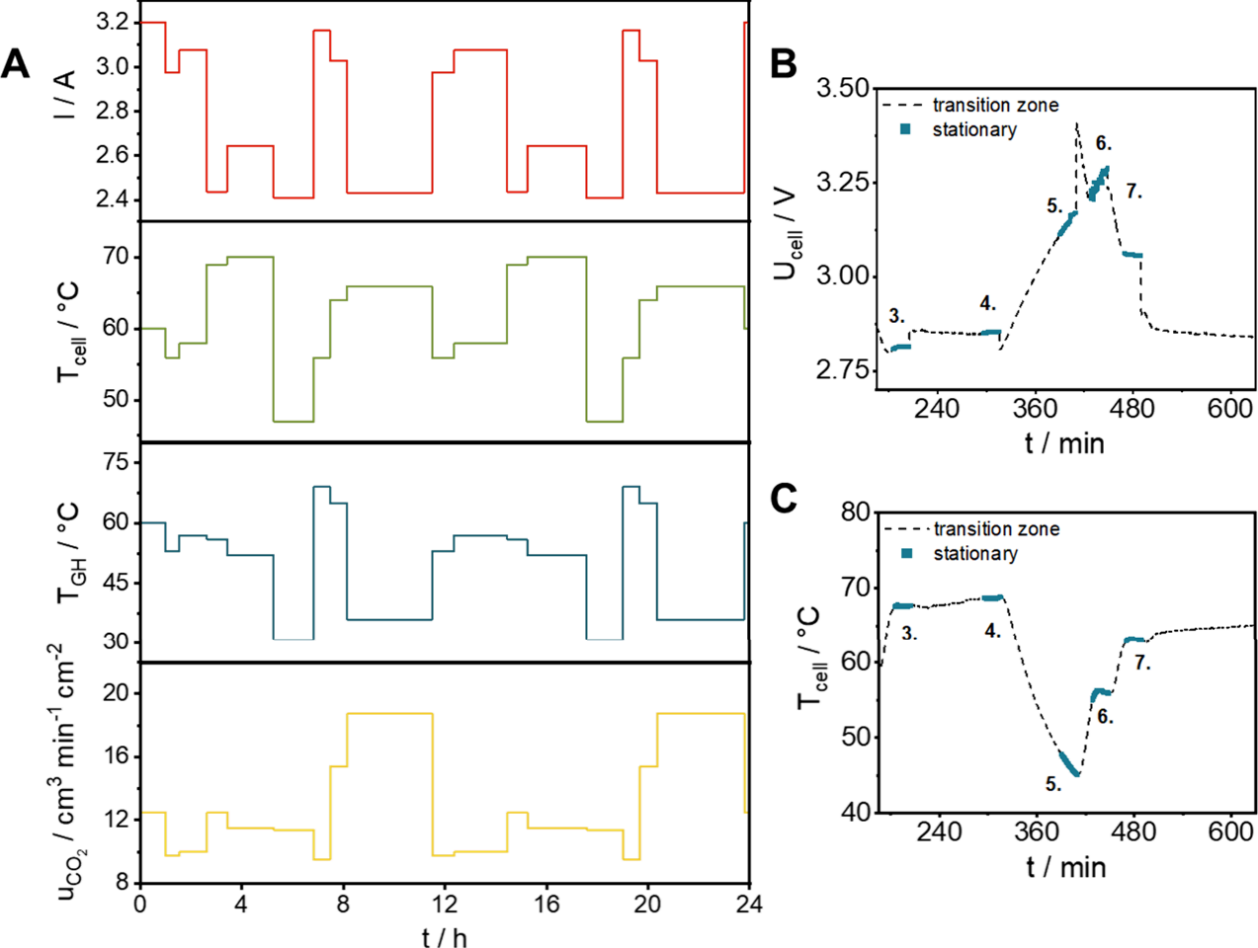
(A) The experimental measurements by running all parameter settings, which was used for model validation (external test set). (B) Typical cell voltage and (C) cell temperature curves recorded during such measurement, indicating short and longer transition times between different experimental settings.

**Figure 4:**
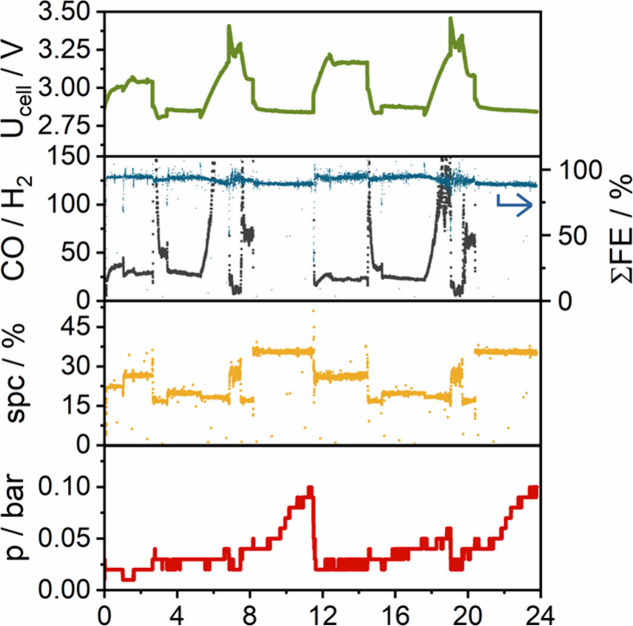
Results gathered when measuring all parameter settings depicted in Figure 3. A CO_2_-saturated 0.05 M CsOH solution was applied as anolyte. SPC denotes single-pass conversion.

Many general notions of the CO2RR community were confirmed when running this test sequence. The cell voltage scales with the applied current and is inversely proportional to the cell temperature. The electrolyzer cell voltage varied between 2.8 – 3.3 V, with a FE­(CO) variation of 90–100%, while the single-pass conversion (SPC) values spanned between 15 and 35%. At lower GH temperatures, CO formation is preferred, resulting in very high selectivity (expressed by the CO/H_2_ ratio). These trends are precisely captured by our sensitivity analysis (see Figure S11), performed on the data set used for model training.

Cathode pressure increase occurred at low GH and cell temperatures, which is attributed to precipitate formation, and its inefficient removal from the cathode gas diffusion electrode.^30^ This further causes a cell voltage increase, which is attributed to the decreasing electrochemically accessible surface area. This notion is supported by the rapid cell voltage decrease when the current is reduced (at ca. 8 h in the data set), although the increased CO_2_ inlet pressure was not affected by this change. Importantly, the pressure increase that is attributed to the precipitate formation did not cause any irreversible damage to the electrolyzer cell-the results gathered when repeating this test sequence (from ca. 12 h) almost completely overlap with those from the first run.

While discussing model prediction accuracy in this section, for practical reasons, we do not include every metric that we used in our experiments (they are shown in Table S3 and Figure S3). Instead, we will present the most interesting findings here, and showcase the predictability of the target features (e.g., cell voltage, product gas composition (V/V% of H_2_, CO, and CO_2_ in the product stream)), and target variables (e.g., Faraday efficiency of CO formation (FE­(CO)), and selectivity). To further support the reliability of the model predictions, a detailed uncertainty analysis was conducted and is presented in the Supporting Information (see Figures S7–S9 and also Tables S4–S7). This analysis quantifies the prediction confidence intervals of the input and target variables within single parameter settings. This offers additional insight into the robustness of the model and compares it to the true results of the measurements.

Quantifying the model prediction accuracy, the trained model predicts the target features with high precision on the test set, even though the model never saw any of these parameter combinations (Figure 2 and Figure S4). Note that the test set was chosen from experiments run on the same electrolyzer cell. If one examines the voltage values predicted by the model, they are closely matched with the actual values of the experiment. This is supported by a low value of *Mean Absolute Error* (MAE) and a high *Coefficient of Determination* (R^2^). *Mean Absolute Percentage Error* (MAPE) is also of low value, showing that the values predicted do not deviate significantly from the true target variable (see all parameters in Table S3). One can reason similarly for the predicted CO, CO_2_, H_2_ concentrations, and the flow rate of the product stream (flow out), as these predictions also indicate high correlation with the target variables. However, FE­(CO) behaves differently. Note that this value is calculated from previous values predicted by the model, so it is affected by prediction errors. Despite this, MAE and MAPE scores show that FE­(CO) could be closely estimated using this model. The R^2^ value is not so encouraging; however, it could be reasoned that FE­(CO) is of high variability with many outlier-like artifacts (Figure 2 and Figure S8). For this reason, the R^2^ value is bound to be lower for this target variable.

The predictive capacity of the developed model was investigated with the parameter settings shown in Figure 3 and Figure 4 (see further details in the Supporting Information, and in Figures S6 and S9). Compared to the performance achieved on the test set, R^2^ values dropped moderately for cell voltage, the amount of CO and CO_2_, and output flow rate. The model performed worse in predicting the amount of H_2_ and FE­(CO). In terms of MAE, there is an order of magnitude increase in all predicted variables compared to the test set. However, this increase is still relatively low if we consider the effect of cell-to-cell variations, typically reported in the field.^31^ MAPE also shows a slight deterioration in model performance for all target variables, most notably for the H_2_ evolution rate. For the rest of the target variables, MAPE increased by only a few percentage points. It should be noted that both FE­(CO) and SPC predictions were less than 5% away from the measured values based on MAPE alone.

As noted above, we believe that the differences in the measured and predicted values are rooted in the cell-to-cell variation, which is represented by error bars in the figures typically used in the CO2RR community (and is much highlighted in the representation used here). While we continue to work on reducing this variation, we also continue to further develop our model and methodology. The latter includes defining and running shorter cell diagnostics measurements, which can be used for “calibrating” the ML model. Note that this would also allow accounting for cell aging, and therefore these tools can also aid long-term CO_2_ electrolysis, under conditions continuously adapted to the current state-of-health of the electrolyzer cell/stack. We consider the implementation of ANN-based methods in the process control architecture as one of the most realistic scenarios for the industrialization of CO2RR.

## Supplementary Material



## Notes

Further information on the ML algorithm used and model training (PDF)
