# Supplementary material for: Neural Algorithm Aided Operation of CO2 Electrolyzers
Source: ACS Energy Lett. 2025 Jul 17;10(8):3845–50. doi: 10.1021/acsenergylett.5c01133 (PMC12341662; doi:10.1021/acsenergylett.5c01133)
Supplement: Supplementary file 1 [file nz5c01133_si_001.pdf]

Supporting Information

Neural Algorithm Aided Operation of CO_2 Electrolyzers

Angelika A. Samu,[1,2,†] Dániel Horváth,[3,†] Balázs Endrődi,[1,] László Vidács,[3] Csaba Janáky[1,2,*]*

[1]Department of Physical Chemistry and Materials Science, University of Szeged, Aradi sq. 1, Szeged, 6720, Hungary

[2]eChemicles Zrt, Alsó Kikötő sor 11, Szeged, 6726, Hungary

[3]Department of Software Engineering, University of Szeged, Szeged, Árpád sq. 2. Szeged, 6720, Hungary

AUTHOR INFORMATION

†(A. A. S., D. H.) These authors contributed equally to this work.

Corresponding Author

*Csaba Janáky, janaky@chem.u-szeged.hu

*Balázs Endrődi, endrodib@chem.u-szeged.hu

Supplementary Methods

Experimental Methods

Preparation of Electrodes

We dispersed 25 mg cm^{-3} Ag nanoparticles (d_{avg} < 100 nm, Sigma-Aldrich) in a 1:1 isopropanol-water solvent mixture to prepare the cathode catalyst ink. This dispersion ink also contained 5 wt% PiperION ionomer (Versogen) as a catalyst binder. The anode catalyst ink contained 20 mg cm^{-3} Ir nanoparticles (d = 4-6 nm, Fuel-Cell Store) in an identical solvent mixture with 15 wt% ionomer content. These dispersions were homogenized for at least 20 minutes in an ultrasonic bath (Elmasonic P 30 H) prior to electrode preparation. We also used a high-power immersion sonotrode (Hielscher UP200ST) to fully disperse the aggregated Ag nanoparticles for 3 minutes and the power of 200 Watt. The cathode and anode catalyst layers were formed by spray-coating, using a hand-held airbrush (Alder AD-320). The dispersions were spray-coated on samples preheated on a hotplate at 100 ˚C, at a constant flow rate. The only difference in the spray coating method was the supporting material, because for the anode catalyst we used a 1 mm thick, porous Ti frit, while carbon-based gas diffusion layers (Sigracet 39BB GDLs) were used as cathode catalyst supports. The cathode and the anode catalyst loadings were 1.0 ± 0.1 mg cm^{-2}. After catalyst coating, we cut into shape with a hollow punch. In all cases, the active surface of the cathode electrode was an 8 cm^2 circle.

Electrolyzer Cell Assembly and Test Framework

A custom-designed direct gas feed zero-gap electrolyzer cell was used for all experiments. The cell consists of two catalyst-coated electrodes, which were separated with only a 40 μm thick anion exchange membrane (PiperION, Versogen). Before use, the membrane was activated by immersing it in a 1.0 M CsOH solution for at least 24 hours. After activation, it was cut into shape using a sharp surgical blade and washed with deionized (DI) water before inserting it into the electrolyzer cell. The electrodes were placed in the cell with the catalyst layers facing the membrane (hence each other as well). Six bolt screws were used to assemble the cell, with a final, gradually applied torque of 3 Nm. The anolyte (0.05 M $CsHCO_3$ solution) was circulated with a peristaltic pump through the anode current collector at a flow rate of 80 $cm^3\ min^{-1}$. Through the cathode current collector, the humidified CO_2 stream was supplied, controlled with a Bronkhorst MASS-STREAM D-6321 type mass-flow controller. A Rohde & Schwarz HMP4040 power supply was applied for the measurements. The composition of the cathode product stream was analyzed with an online infrared-thermal conductivity gas analyzer (Gasboard-3100, customized for CO_2-CO-H_2 mixtures, Hubei Cubic-Ruiyi), which allowed a real-time determination of the product composition.

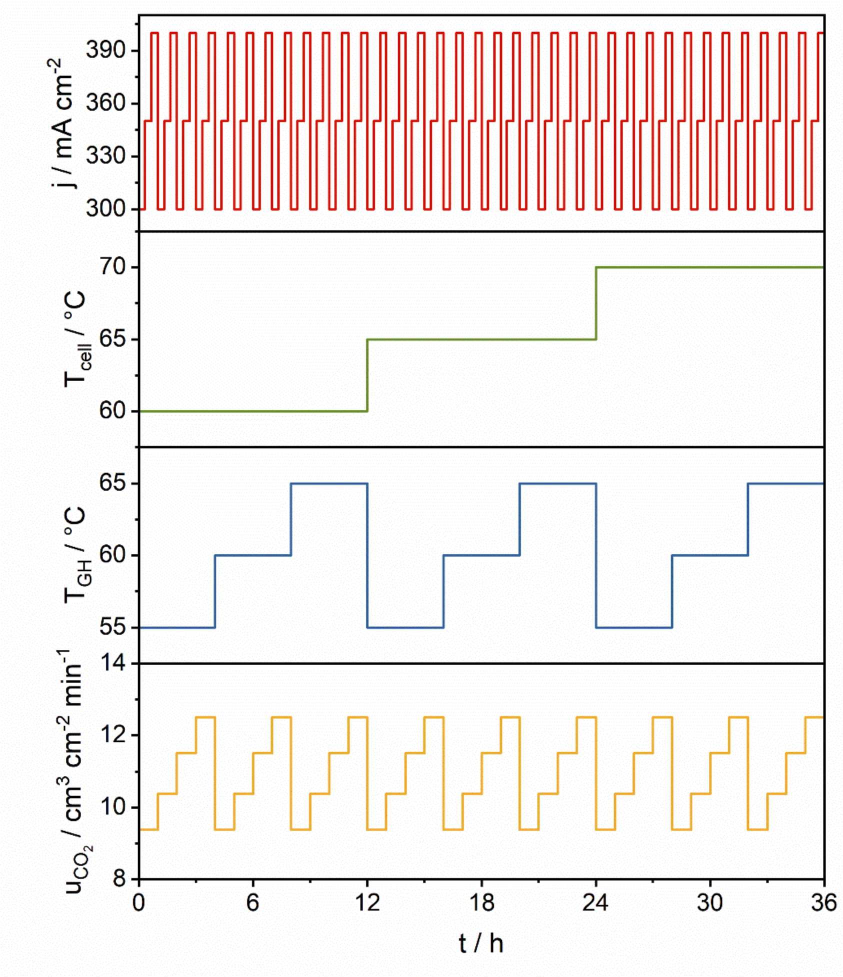

Figure S1. Parameter settings for electrolysis experiments.

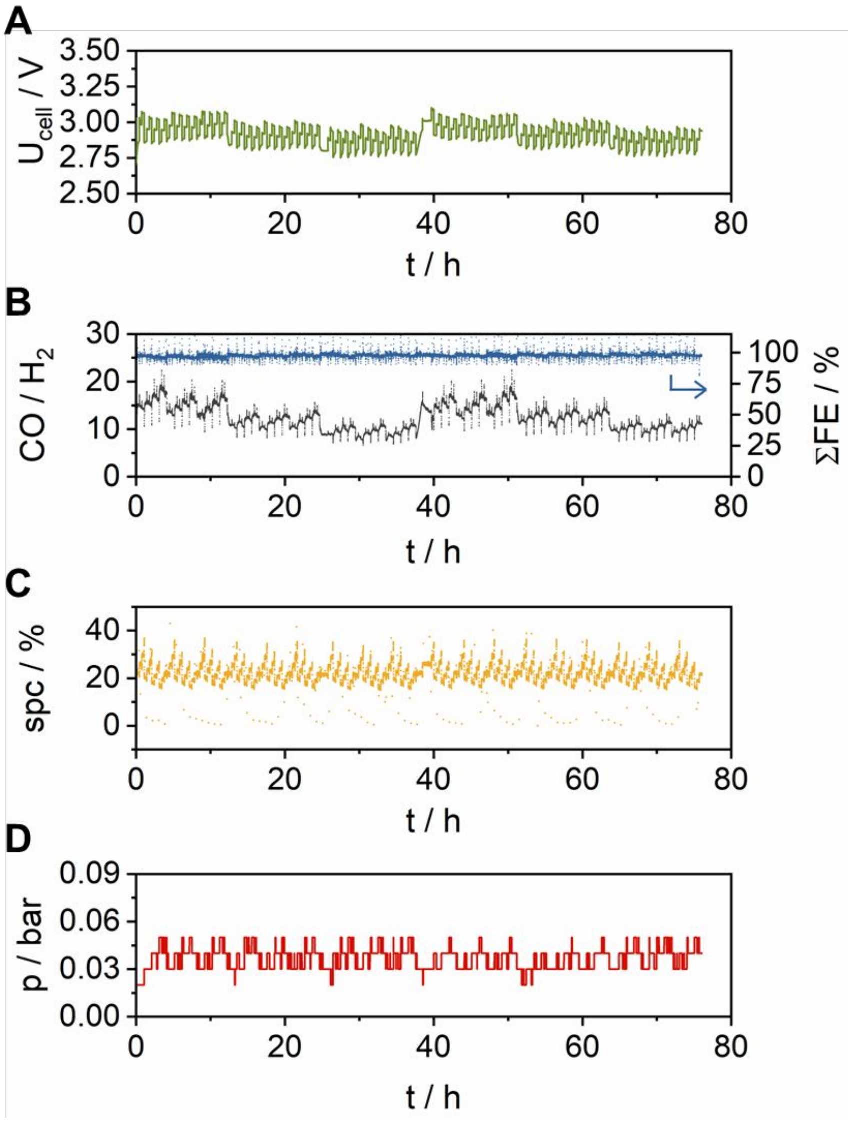

Figure S2. Results gathered during electrolysis measurements at 108 different parameter combinations (shown in **Figure S1**., repeated twice in a row), performed for model development: (A) cell voltage, (B) selectivity, (C) single-pass conversion (spc) and (D) the pressure measured upstream of the electrolyzer cell. A 0.05 M $CsHCO_3$ solution was applied as anolyte.

Data Structure

To better explain the difference between a single data point and a measurement sequence (i.e. parameter combination), we provide the following example in **Table S1**. Shortly, data was accumulated for 1200 s with 5 s intervals at each parameter setting. In the data splitting step, the individual data points are already handled in measurement sequences (as groups) and moved to the train, validation and test sets accordingly. This data handling strategy prevents potential data leaks and ensures a fair evaluation of the models. All the data points recorded for a single parameter setting were evaluated together, adding up to 240 readings per parameter setting (which might be decreased during data filtering in case faulty readings are observed).

Table S1. Simplified illustration of the dataset format used during this study. The double-line-styled border signifies the switch between parameter settings. In this case, Set current , and Set GH temp are two examples of control values, with their corresponding measured values of Current , and GH temp , respectively. The measured target values in each row can also be observed (note the possible fluctuations in value) as Voltage, CO, CO_2, and H_2 .

#	Set current / A	Set GH temp / °C	Measured Current / A	GH temp. / °C	...	Voltage / V	CO V/V%	CO_2 V/V%	H_2 V/V%	...
...	...	...	...	...	...	...	...	...	...	...
i	2.4	60	2.401	61.2	...	2.701	26.57	71.23	3.67	...
i+1	2.4	60	2.402	61.1	...	2.712	26.98	71.98	3.65	...
i+2	2.4	60	2.399	60.8	...	2.694	26.38	71.65	3.64	...
i+3	2.8	55	2.800	56.1	...	3.012	31.98	67.98	2.87	...
i+4	2.8	55	2.801	56.0	...	3.101	31.13	67.65	2.67	...
i+5	2.8	55	2.799	55.7	...	3.087	31.74	67.39	2.78	...
i+6	2.8	55	2.799	55.6	...	3.059	31.46	68.43	2.97	...

...	...	...	...	...	...	...	...	...	...	...

To end this section, we include some technical details and naming conventions used throughout this paper, to better understand the meaning behind each wording of feature names presented in this paper.

Table S2. Correspondence between control- and model input features.

Control Variable	Input Feature
Current Limit Setpoint [A]	Current [A]
Cell Temp Setpoint [deg C]	Cathode Temp [deg C]
GH Temp Setpoint [deg C]	GH Temp [deg C]
MFC Setpoint [sccm]	Flow [sccm]

In this case the control variables are settings that can be set during the measurement phase. These settings are validated by the system by measuring them in real time and also reporting the true values of these variables. Our models use the column of input features as their training data, with every sample as a different measurement point.

We also use some aliases for some of the output values. For example, for the Faraday Efficiency we often use faradaic efficiency or *FE(CO) [%]*, and we also use *CO_2 conversion rate [%]* and *SPC [%]* interchangeably, often out of necessity for space (e.g., in tables).

Model Selection Experiments

Selecting a baseline model for our case study was not a trivial task, as the number of ML algorithms is continuously increasing, with newer and more sophisticated strategies. It is outside the scope of this study to list all possible algorithms to choose from; however, we did conduct a series of experiments to get insight into the intricacies of different models. While conducting these experiments, our goal was to select the

predictive model that would offer the best trade-off between accuracy and generalizability across different cell assemblies, also keeping in mind the ease of use and the possibility to further upgrade the selected model.

Table S3. Artificial Neural Network, Random Forest Regressor, Linear Regressor, and XGBoost Regressor model predictions on the test, and external test set.

	Test set					External test set				
	R^2	MSE	RMSE	MAE	MAPE	R^2	MSE	RMSE	MAE	MAPE
	**Artificial Neural Network**									
**Voltage [V]**	0.9892	9.6347E-05	9.8157E-03	7.8167E-03	2.5955E-03	0.8184	5.0718E-03	7.1216E-02	5.6806E-02	1.8082E-02
**CO [%]**	0.9960	1.8197E-01	4.2658E-01	3.1806E-01	1.0431E-02	0.9119	6.4593E+00	2.5415E+00	1.6839E+00	6.7276E-02
**CO_2 [%]**	0.9963	1.6060E-01	4.0075E-01	3.0577E-01	5.2516E-03	0.7823	1.1114E+01	3.3338E+00	2.4628E+00	3.8523E-02
**H_2 [%]**	0.9843	1.8863E-02	1.3734E-01	9.9188E-02	4.3357E-02	0.0455	1.0530E+00	1.0262E+00	9.7229E-01	1.5113E+14
**Flow out [sccm]**	0.9943	3.2587E+00	1.8052E+00	1.3539E+00	1.1354E-02	0.9614	7.4411E+01	8.6262E+00	5.0597E+00	3.8184E-02
**FE(CO) [%]**	0.5356	1.8961E-04	1.3770E-02	9.9178E-03	1.1313E-02	-3.5247	2.2081E-03	4.6991E-02	3.9980E-02	4.4964E-02
**SPC [%]**	0.9904	1.1653E-05	3.4136E-03	2.4724E-03	1.1313E-02	0.9593	1.0702E-04	1.0345E-02	8.9142E-03	4.4964E-02
**Average**	0.9266	5.1720E-01	3.9955E-01	2.9959E-01	1.3659E-02	0.1363	1.3292E+01	2.2366E+00	1.4692E+00	4.1999E-02
	**Random Forest Regressor**									
**Voltage [V]**	0.9726	2.4340E-04	1.5601E-02	1.1846E-02	3.8987E-03	0.5076	1.3756E-02	1.1729E-01	8.3694E-02	2.6422E-02
**CO [%]**	0.9949	2.3293E-01	4.8263E-01	3.6682E-01	1.2029E-02	0.8407	1.1679E+01	3.4174E+00	2.5349E+00	1.1545E-01
**CO_2 [%]**	0.9969	1.3354E-01	3.6543E-01	2.7229E-01	4.6328E-03	0.7234	1.4122E+01	3.7579E+00	2.7860E+00	4.3569E-02
**H_2 [%]**	0.9442	6.7066E-02	2.5897E-01	1.8267E-01	7.5441E-02	-0.4426	1.5914E+00	1.2615E+00	1.1980E+00	4.5967E+14
**Flow out [sccm]**	0.9944	3.2141E+00	1.7928E+00	1.2682E+00	1.0606E-02	0.5577	8.5238E+02	2.9195E+01	1.5493E+01	8.3024E-02
**FE(CO) [%]**	0.3100	2.8175E-04	1.6786E-02	1.2812E-02	1.4593E-02	-2.7019	1.8066E-03	4.2504E-02	3.3550E-02	3.7752E-02
**SPC [%]**	0.9847	1.8647E-05	4.3182E-03	3.2579E-03	1.4593E-02	0.9609	1.0300E-04	1.0149E-02	7.8817E-03	3.7752E-02
**Average**	0.8854	5.2117E-01	4.1950E-01	3.0255E-01	1.9399E-02	0.0637	4.5679E+00	5.4003E+00	3.1624E+00	5.7329E-02
	**Linear Regressor on 2nd order polynomial features**									
**Voltage [V]**	0.9791	1.8557E-04	1.3622E-02	1.0138E-02	3.3645E-03	0.7776	6.2122E-03	7.8818E-02	6.4854E-02	2.0647E-02
**CO [%]**	0.9959	1.8858E-01	4.3425E-01	3.1341E-01	1.0103E-02	0.7232	2.0288E+01	4.5042E+00	2.2266E+00	1.2630E-01
**CO_2 [%]**	0.9954	2.0108E-01	4.4842E-01	3.5982E-01	6.0944E-03	-0.1793	6.0203E+01	7.7591E+00	4.0991E+00	6.0248E-02
**H_2 [%]**	0.9695	3.6681E-02	1.9152E-01	1.3175E-01	5.4210E-02	-1.7186	2.9992E+00	1.7318E+00	1.3298E+00	1.6309E+15
**Flow out [sccm]**	0.9939	3.4739E+00	1.8638E+00	1.4171E+00	1.1833E-02	0.9785	4.1364E+01	6.4315E+00	4.3075E+00	2.8344E-02
**FE(CO) [%]**	0.6046	1.6147E-04	1.2707E-02	9.3193E-03	1.0615E-02	-288.1289	1.4110E-01	3.7563E-01	1.3007E-01	1.4735E-01

**SPC [%]**	0.9911	1.0840E-05	3.2923E-03	2.3700E-03	1.0615E-02	0.1647	2.1978E-03	4.6880E-02	1.9242E-02	1.4735E-01
**Average**	0.9328	5.5722E-01	4.2395E-01	3.2055E-01	1.5262E-02	-41.0547	1.7858E+01	2.9897E+00	1.7396E+00	8.8375E-02
	**XGBoost**									
**Voltage [V]**	0.9826	1.5467E-04	1.2437E-02	9.7584E-03	3.2270E-03	0.4073	1.6556E-02	1.2867E-01	1.0201E-01	3.2380E-02
**CO [%]**	0.9932	3.1466E-01	5.6094E-01	3.5828E-01	1.1500E-02	0.8937	7.7882E+00	2.7907E+00	2.3703E+00	1.0600E-01
**CO_2 [%]**	0.9950	2.1749E-01	4.6636E-01	2.7687E-01	4.6876E-03	0.8942	5.4002E+00	2.3238E+00	1.7454E+00	2.6027E-02
**H_2 [%]**	0.9590	4.9297E-02	2.2203E-01	1.5148E-01	6.3985E-02	-0.0883	1.2006E+00	1.0957E+00	1.0581E+00	4.7134E+14
**Flow out [sccm]**	0.9927	4.1835E+00	2.0454E+00	1.2760E+00	1.0954E-02	0.6012	7.6864E+02	2.7724E+01	1.4308E+01	7.5049E-02
**FE(CO) [%]**	0.4773	2.1345E-04	1.4610E-02	1.1122E-02	1.2654E-02	-6.7081	3.7617E-03	6.1333E-02	5.2848E-02	5.8807E-02
**SPC [%]**	0.9883	1.4240E-05	3.7736E-03	2.8300E-03	1.2654E-02	0.9107	2.3506E-04	1.5332E-02	1.2952E-02	5.8807E-02
**Average**	0.9126	6.8077E-01	4.7507E-01	2.9805E-01	1.7095E-02	-0.4413	2.4016E+00	4.8771E+00	2.8071E+00	5.9511E-02

Several model types were considered ranging from simpler linear regression models to random forest regressors, XGBoost and Artificial Neural Network (ANN) models. All of the models performed surprisingly well on the test set (see **Table S3**); however, they mostly failed on the external set (except the ANN), especially in terms of the average R^2 value. In this sense, Random Forest Regressor is the only model (apart from ANN) that is able to produce a positive value on the average R^2 metric (see **Table S3**). This implies that most of the models fail to generalize well on different cell assemblies.

Figure S3 provides a visual comparison of the average performance of the ANN and the alternative models, on the test and external test sets. Note that the hue of the last rows of **Table S3** matches the color of the average metric value presented in the figure. Another notable piece of information in the table is the grey color of cells for the predicted *H_2 [%]* value. These values are outliers that are mainly the result of the sensitivity limitations of the gas analyzer and data preprocessing. In cases where the amount of H_2 in the output gas decreased below the sensitivity threshold of the gas analyzer it returned unrealistically high values. These faulty values – as mentioned in the m-s – are filtered out by replacing them with zero. As a result of this data preprocessing step, the MAPE metric increases unrealistically, and as such, we left out

these values in the case of the average calculations. Even with this allowance in mind, MAPE still performed significantly worse (2-4 times) on the external test set for all used algorithms.

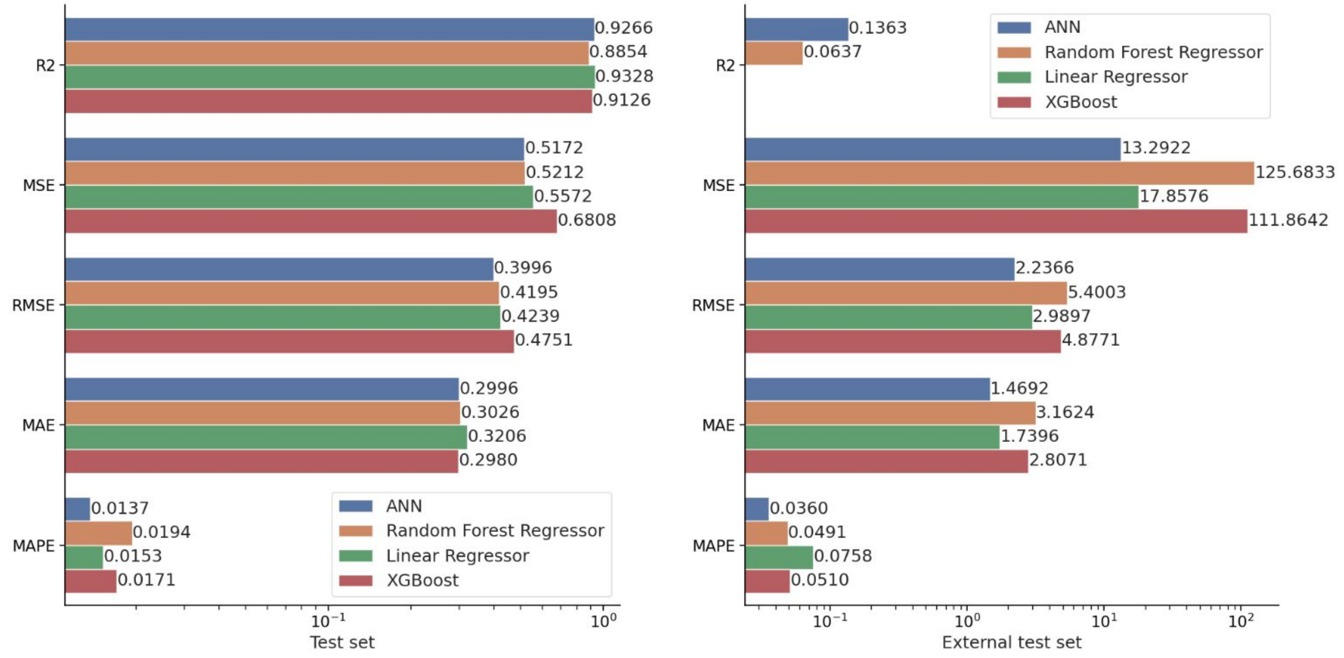

Figure S3. Illustration of the overall model performance averaged over all separate output values on the test set and the external test set. The y-axis shows the different metrics used to evaluate the fitness of the model, while the x-axis shows the values of these metrics. The different colors help to distinguish between the various models and their performance. Note that, for practical purposes, we used a log-scale on the x-axis, as some values could show notable differences. The values of the metrics are also shown to aid in the comparison of performances. On the external test set, the R^2 values for the Linear Regression and XGBoost models are negative, implying poor correlation. The negative values are replaced by 0 and not shown in the plot, due to the logarithmic scaling of the x-axis.

Ultimately, a relatively simple Artificial Neural Network (ANN) architecture emerged as the most effective model for our experiments. The chosen model achieved the best test results among other ML-based methods

and provided a strong balance between flexibility and generalization. Also, this model was proven to be the most robust in terms of predictive capacity across different cell assemblies.

In conclusion, while a variety of modelling techniques were evaluated, the ANN model provided the best overall performance and generalizability across different cells. The models seen in **Table S3** are documented for reference and comparative purposes, but ultimately, they were not selected for the final optimization processes.

Model Evaluation on the Test Set

Figure S4 shows the evaluation of the ANN model on the test set. It can be deduced that the model performs well, based on the shown R^2 values, which basically tells us how well a statistical model predicts the dependent variables based on the given independent variables. Normally, the closer the R^2 value is to 1, the better the model's prediction prowess.

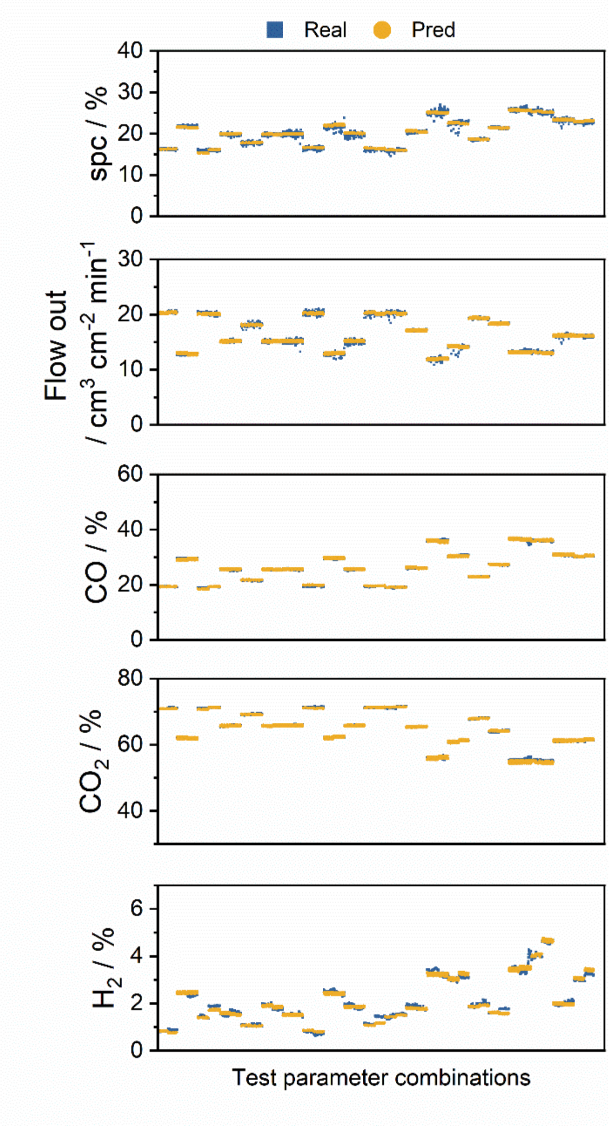

Figure S4. Illustration of the validity of the developed model on the test set, in addition to what is shown in **Figure 2** in the m-s. The model was trained via the measurement sequence built from varying experimental parameters according to **Figure S1** and **Figure S2**. The test set was 20% of the total number of parameter settings (with all the data points belonging to the given parameter settings), carefully separated from the rest without any overlap.

Parameter Optimization

In the parameter optimization step, the goal was to test the validity of the model's predictions in an extended parameter space and find the optimal settings for the electrochemical cell. We used *Optuna*'s Python package to guide this process.[1] We note, that the source code of the Optuna implementation of the NSGA-II algorithm is readily available here: https://github.com/optuna/optuna/tree/master/optuna/samplers/nsgaii

The maximum number of trials (the overall budget) was set to 10,000. The optimization metrics were set to be *FE(CO) [%]* and *CO_2 Conversion [%]*, while also optimizing for *Cell Voltage [V]*. The first two values were set for maximization, while the Cell Voltage was minimized. When optimizing for multiple targets, *Optuna* defaults to using the so-called Non-dominated sorting genetic algorithm (NSGA-II)[2] with its default parameter settings of population size of 50, a crossover probability of 90%, and a mutation probability of 1 divided by the length of trial parameters to be optimized (3 in our case).

In the optimization process, the trained model was employed to predict target variables for thousands of previously – highly likely – unseen input variable combinations. Unlike single-objective optimization, multi-objective optimization aims to find solutions that have the best trade-offs between different objectives. The set of these solutions is also known as the Pareto front, where it can be said that improving one objective leads to the degradation of others. In other words, none of the solutions can be said to be better than the other. The NSGA-II algorithm consists of 8 main steps:

1. Initialization of the first population
2. Fitness evaluation
3. Non-dominated sorting
4. Calculation of crowding distance
5. Selection of parents
6. Application of genetic operators (crossover, mutation)
7. Creation of the next generation (using elitism)
8. Process termination if the criteria are met

To better understand the sequence of steps in NSGA-II, **Figure S5** provides a visual explanation of the algorithm via a flow chart.

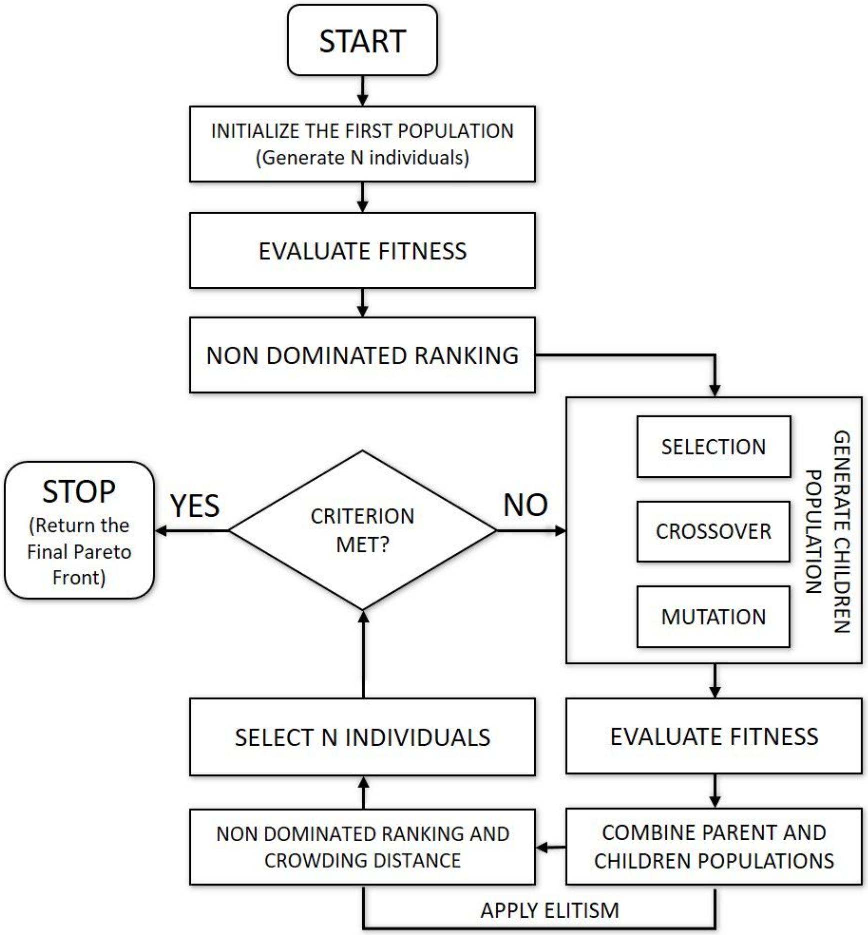

Figure S5. Flow chart of the NSGA-II algorithm. The design of the flow chart is inspired by ref. [3].

When the optimization process was over and all 10,000 trials were run successfully, we selected 8 different parameter sets provided by the selection procedure. When selecting these parameters, a significant selection criterion was to include 4 parameter combinations from the lower-end optimization trials, and 4 from the higher-end trials, while also considering that the selected combinations should be of different settings, if possible. This meant that we would have 8 distinct parameter combinations, which are considerably different from each other, for the external test set.

Model Evaluation on the External Test Set

For the external test set, data collection was performed on another but otherwise identical cell assembly to the one we used for the development of the model. The parameter setting with the highest gas flow rate (150 sccm) was invalidated in the first measurement, as this parameter combination never settled in a stable state rendering it unreliable. The second trial was kept, as it may contain useful information on cell behavior outside the typical parameter settings. Note that, for this reason, only 15 parameter settings remained after all the data pre-processing steps, keeping two repetitions for 7 of the parameter combinations and one single measurement for 1 parameter combination.

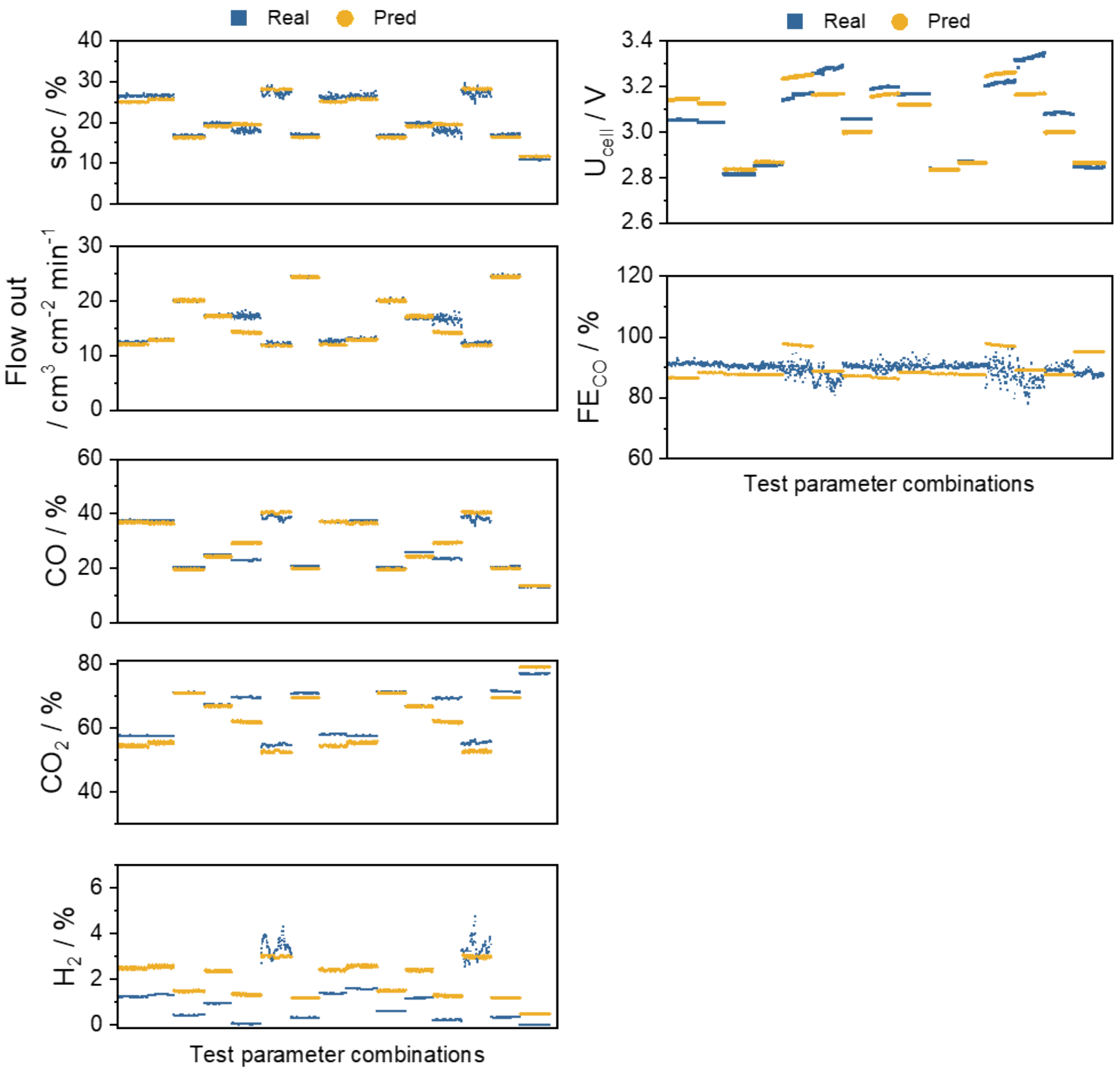

Figure S6. Illustration of the validity of the model, tested on the external test set shown in **Figure 3** and **4**. in the m-s.

By examining **Figure S6**, or **Table S3** for overall metrics and **Table S6** for comparison of mean values of the predictions and the mean of the measured values, we can conclude that the model does in fact lose some of its performance when applied to a different cell assembly. However, some of the values keep close to their original performance. For example, *CO [%]* and *Flow out [sccm]* give above 0.9 value for R^2 with MAPE values of 0.0673 and 0.0382, indicating a good predictive capability. Voltage and CO_2 are predicted moderately well compared to other values such as H_2. Among the derived values, single-pass conversion is explained well by the model according to the R^2 metric, and a MAPE score of 0.0496 also shows a low error

rate in the calculated value. A low error rate can also be observed in the calculated value of *FE(CO) [%]*, however, due to the already high variance of this value, R^2 shows significant degradation. Because of its high variance –even in the training data– R^2 alone should not be used for judging model performance, hence the inclusion of other metrics, to complement each other.

Overall, it is our conclusion that even for different cell assemblies, some of the values can be predicted more efficiently (e.g. Flow out, CO, and CO_2), while others are affected more negatively (H_2, and FE(CO)) by applying the same model on a different assembly.

Uncertainty Analysis

In this section we analyze the uncertainty in the measured input features during different parameter settings, so we can provide insights into the stability of these specific measurements. We also analyze these uncertainties in the model output, compared to the true measured values so that we can analyze possible differences and similarities between the learned system and the behavior of the data provided by the measurements.

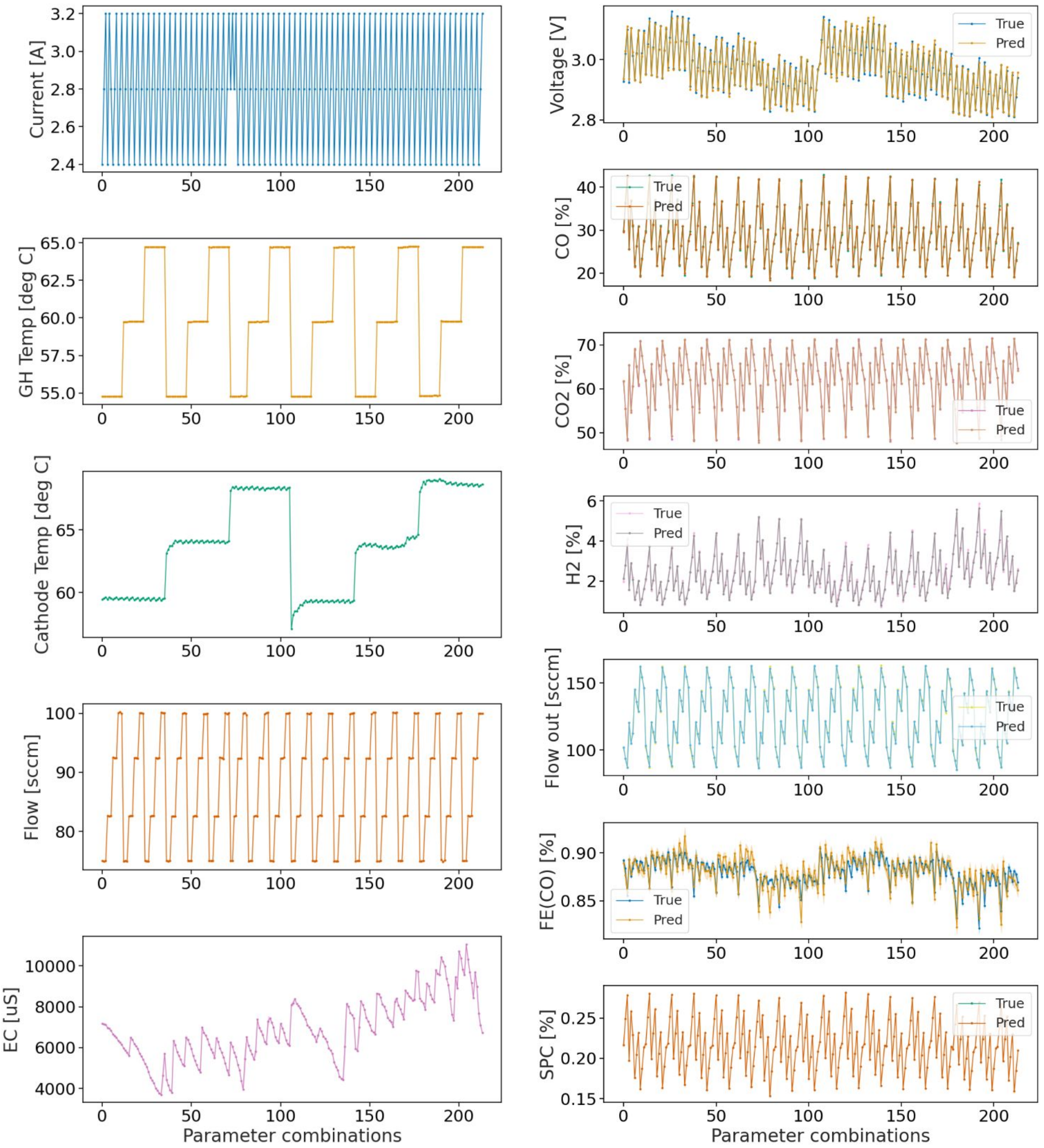

Figure S7. Representing uncertainty in the data and the model predictions on the full development dataset. On the left side, means and standard deviations are shown for the input features, while on the right, the output features can be observed for the ANN predictions, and for the true values as well. Note that this figure

contains all the parameter combinations used for the development of the model, hence model predictions for the train, validation and the test sets are also included.

As every parameter setting has multiple measurement points, it is reasonable to assume some level of uncertainty. While we attempted to minimize the variance in the input and output features, some deviation from the measurement sequence mean is expected. If one examines **Figure S7** it is apparent that the cell operation for input features, as well as the targeted output features could be considered stable. We plotted the mean of every parameter setting (ordered by time) and drawn the full scale of data spread (confidence interval of 100%) for every setting which is depicted by a band surrounding the mean value. The lines connecting the dots only help to visualize the ordering of the different measurement sequences. One can see that the largest uncertainty is associated with FE(CO). Note, that FE(CO) is calculated from the gas flow rate and the gas composition, and therefore the uncertainty of these two is reflected in this together. To better understand the behavior of the model on previously unseen data, **Figure S8** visualizes the uncertainty in the data and in the predictions of the model on the test set.

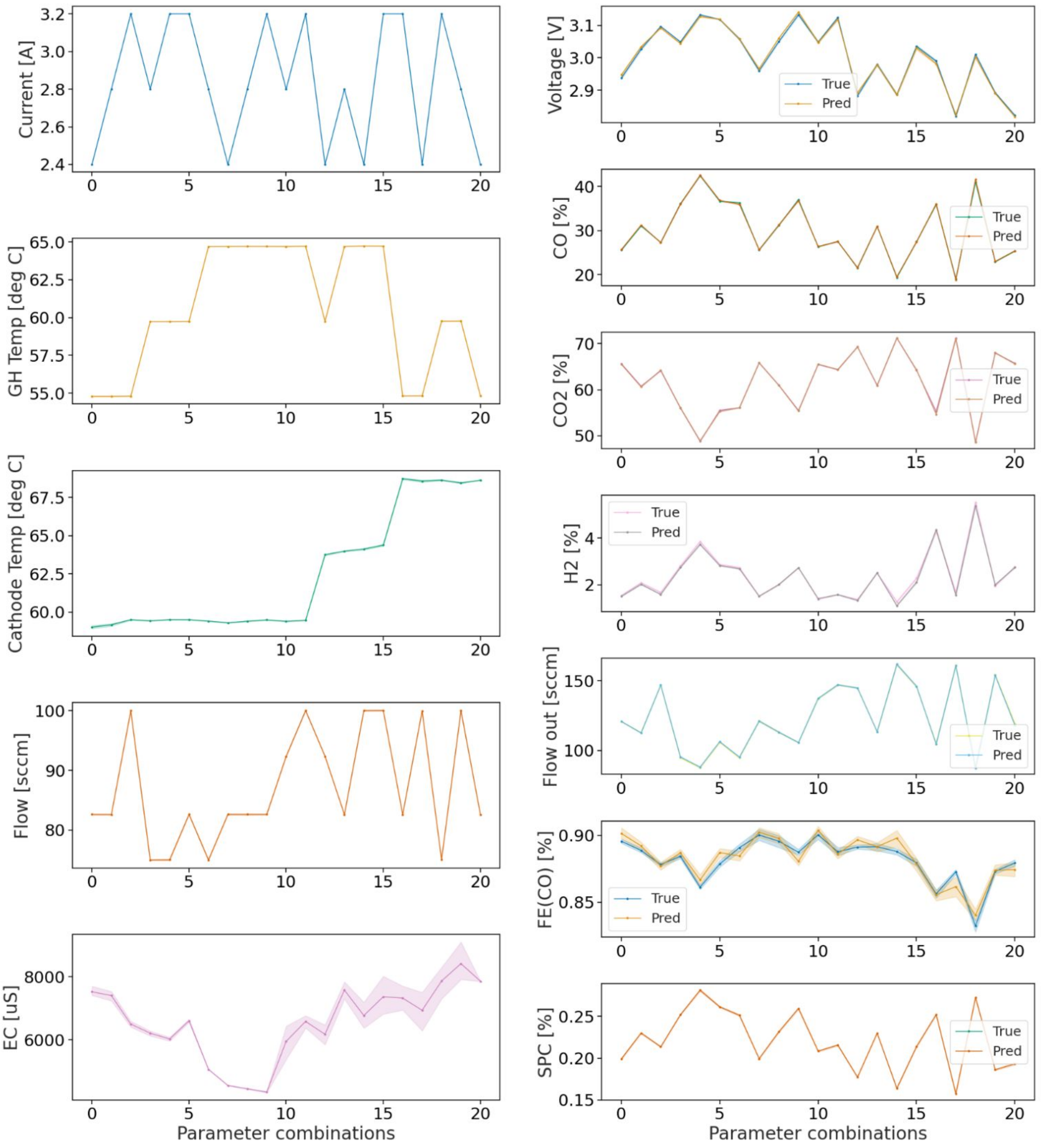

Figure S8. Representing uncertainty in the data and the model predictions on the test set. The left side shows the mean and the confidence interval for the input values, while on the right side one can observe these values for the ANN predictions, and the true values for the test set.

The stability of input and output values is comparable to the full dataset. To see the numerical representation of these values, see **Table S4**.

Table S4. Comparing ANN predicted and true mean values for individual measurement sequences for each parameter setting on the test set. For every two rows found on the right side there is a single corresponding input setting, which is the measured mean value of the specific settings. The first row of every pair represents the true (measured) mean value of the output features, while the second row is the model's predicted mean value. Values with light blue color indicate lower values for that particular column or feature, with darker blue indicating values in the middle range, and red indicating values in the higher range of that setting. The purpose of coloring is twofold: for once, they indicate where to search for parameters that are possibly outside the range of the trained parameter settings, and two, they are comparable by pair, meaning that similar if for one index two colors are similar in shade, they are also similar in value. The last row (averaged distance of values) indicates the averaged distance between every pair of values per column, showing how far are the prediction means from the true measurement means on average. EC stands for the conductance of the anolyte.

#	Current [A]	GH Temp [deg C]	Cathode Temp [deg C]	Flow [sccm]	EC [uS]	Voltage [V]	CO [%]	CO2 [%]	H2 [%]	Flow out [sccm]	FE(CO) [%]	CO2 Conversion Rate [%]	Type
0	2.3999	54.7839	59.0135	82.6180	7522	2.9381	25.5498	65.6332	1.5529	120.7890	0.8956	0.1979	True mean
						2.9474	25.6938	65.5685	1.5110	121.0358	0.9027	0.1995	Pred mean
1	2.8000	54.7850	59.1666	82.6096	7403	3.0266	31.0339	60.7712	2.0910	112.9085	0.8887	0.2291	True mean
						3.0341	31.2103	60.6492	2.0228	112.6347	0.8918	0.2299	Pred mean
2	3.2000	54.7994	59.5004	99.9905	6499	3.0966	27.3223	64.1120	1.6822	146.7665	0.8781	0.2138	True mean
						3.0919	27.3037	64.2156	1.5923	146.6598	0.8766	0.2134	Pred mean
3	2.8000	59.7442	59.4294	74.9916	6210	3.0489	36.0879	56.0105	2.8098	94.8761	0.8844	0.2512	True mean
						3.0441	36.0229	56.0892	2.7452	95.4325	0.8875	0.2521	Pred mean
4	3.2000	59.7446	59.5035	75.0121	6024	3.1322	42.4359	48.8019	3.8541	87.7649	0.8613	0.2795	True mean
						3.1273	42.5739	48.8851	3.7374	88.1624	0.8680	0.2817	Pred mean

5	3.2000	59.7550	59.5006	82.6190	6602	3.1178	36.6363	55.5486	2.8712	105.9065	0.8786	0.2589	True mean
						3.1178	36.7874	55.2810	2.8216	106.3948	0.8866	0.2612	Pred mean
6	2.7999	64.7010	59.4082	74.9840	5064	3.0575	36.3274	56.0876	2.7381	94.8841	0.8908	0.2530	True mean
						3.0585	35.9744	56.1189	2.7014	95.4574	0.8863	0.2517	Pred mean
7	2.3999	64.7081	59.3007	82.6231	4551	2.9602	25.5880	65.8787	1.5306	121.2194	0.9002	0.1989	True mean
						2.9661	25.6424	65.8329	1.5159	121.1265	0.9014	0.1992	Pred mean
8	2.7999	64.7107	59.4002	82.6168	4445	3.0498	31.1821	60.9995	2.0195	113.2137	0.8956	0.2309	True mean
						3.0605	31.2528	60.9363	2.0143	113.0953	0.8968	0.2312	Pred mean
9	3.2000	64.7111	59.4868	82.6275	4343	3.1324	37.0418	55.5169	2.7294	105.7001	0.8874	0.2614	True mean
						3.1387	36.7045	55.3226	2.7361	105.6883	0.8782	0.2587	Pred mean
10	2.7999	64.7069	59.3946	92.3537	5949	3.0482	26.3256	65.4573	1.3949	137.2202	0.9004	0.2076	True mean
						3.0469	26.4475	65.5287	1.4253	137.1653	0.9046	0.2086	Pred mean
11	3.2000	64.7217	59.4608	100.0275	6582	3.1236	27.5404	64.3591	1.5883	147.1229	0.8877	0.2160	True mean
						3.1177	27.4615	64.3710	1.5827	147.0029	0.8842	0.2152	Pred mean
12	2.3998	59.7509	63.7540	92.3702	6174	2.8833	21.4951	69.3044	1.3796	144.8661	0.8912	0.1761	True mean
						2.8923	21.6096	69.2850	1.3376	144.8602	0.8961	0.1771	Pred mean
13	2.8000	64.7055	63.9909	82.6029	7577	2.9802	30.9553	60.9871	2.5093	113.4196	0.8915	0.2299	True mean
						2.9784	30.9687	60.9038	2.5200	113.4505	0.8921	0.2300	Pred mean
14	2.3999	64.7409	64.1268	100.0275	6770	2.8868	19.3681	71.1839	1.2796	161.3731	0.8879	0.1620	True mean
						2.8873	19.5332	71.2755	1.1188	162.0395	0.8992	0.1641	Pred mean
15	3.2000	64.7368	64.3821	100.0449	7365	3.0354	27.4858	64.1850	2.2839	145.7576	0.8795	0.2140	True mean
						3.0307	27.5521	64.3464	2.1190	146.1186	0.8835	0.2150	Pred mean
16	3.2000	54.8106	68.7270	82.6119	7323	2.9901	35.8981	55.3081	4.3010	105.1073	0.8563	0.2523	True mean
						2.9824	36.1056	54.6129	4.3652	104.4795	0.8568	0.2524	Pred mean
17	2.3999	54.8173	68.5787	99.9687	6936	2.8201	19.0659	71.2177	1.6772	161.0919	0.8728	0.1594	True mean
						2.8249	18.8137	70.9533	1.5630	160.4833	0.8570	0.1565	Pred mean
18	3.2000	59.7631	68.6403	75.0460	7862	3.0110	41.0121	48.7109	5.5104	87.6827	0.8323	0.2700	True mean
						3.0017	41.5202	48.4671	5.3837	87.1701	0.8385	0.2720	Pred mean
19	2.7999	59.7711	68.4586	100.0002	8417	2.8931	22.9297	67.9457	1.9634	154.1893	0.8731	0.1859	True mean
						2.8914	23.0267	67.9881	2.0177	153.7816	0.8749	0.1863	Pred mean
20	2.3999	54.8150	68.6351	82.6113	7860	2.8234	25.3383	65.7571	2.7785	119.1888	0.8793	0.1943	True mean
						2.8199	25.4433	65.6282	2.7296	118.4574	0.8777	0.1940	Pred mean
**Column mean**						3.0028	29.8635	61.5723	2.3835	122.8987	0.8822	0.2212	
**Averaged distance of values**						0.0050	0.1542	0.1366	0.0627	0.3330	0.0048	0.0012	

Based on the colorings, and the last row that shows the average distance of the predicted means from the true values, it can be said that the model predictions are indeed close to the true values measured on the test station. Note that these parameter combinations have never been seen during the training phase, however, they were produced by the same cell configuration as the model was trained on. Based on the average distances we can conclude that the ANN model captures the behavior of a particular cell configuration

relatively well as these values are close to zero. For example, the largest average distance (0.3330) can be observed at the *Flow out* column, however, this is still less than 1 % of the column mean (122.89). We reason that while 0.3330 is indeed the largest of the average distances, it is still close to the real values relative to the scale of the *Flow out* values. The most significant difference (~2.6%) in this sense is observed in the prediction of H_2 value, which has a column mean of 2.3835, and an average distance of 0.0627.

Table S5. Comparing ANN predicted and true std values for individual measurement sequences for each parameter setting on the test set.

#	Current [A]	GH Temp [deg C]	Cathode Temp [deg C]	Flow [sccm]	EC [uS]	Voltage [V]	CO [%]	CO2 [%]	H2 [%]	Flow out [sccm]	FE(CO) [%]	CO2 Conversion Rate [%]	Type
0	0.0002	0.0298	0.4755	0.4780	580	0.0157	0.0893	0.2656	0.0389	0.8067	0.0068	0.0019	True std
						0.0135	0.3638	0.4191	0.0972	1.6048	0.0155	0.0036	Pred std
1	0.0002	0.0297	0.3813	0.4746	612	0.0147	0.1049	0.2458	0.1060	0.7911	0.0075	0.0023	True std
						0.0109	0.4307	0.3877	0.0990	1.5168	0.0133	0.0038	Pred std
2	0.0002	0.0230	0.1080	0.5013	359	0.0011	0.2502	0.2430	0.0931	0.9727	0.0055	0.0018	True std
						0.0064	0.4081	0.4006	0.0702	1.9901	0.0176	0.0044	Pred std
3	0.0002	0.0177	0.1067	0.5028	342	0.0041	0.3609	0.2989	0.1064	1.0263	0.0085	0.0027	True std
						0.0053	0.5243	0.4933	0.0821	1.5295	0.0131	0.0038	Pred std
4	0.0001	0.0378	0.1166	0.5015	272	0.0059	0.4080	0.3507	0.1213	1.0636	0.0085	0.0032	True std
						0.0067	0.6581	0.6636	0.1132	1.6011	0.0154	0.0053	Pred std
5	0.0002	0.0205	0.1038	0.4900	203	0.0058	0.3992	0.3366	0.1012	1.3143	0.0106	0.0035	True std
						0.0067	0.5610	0.4923	0.0883	1.5353	0.0152	0.0049	Pred std
6	0.0003	0.0233	0.0860	0.5080	30	0.0159	0.3839	0.2851	0.1124	1.3551	0.0124	0.0038	True std
						0.0062	0.5691	0.5606	0.0908	1.6494	0.0137	0.0041	Pred std
7	0.0002	0.0292	0.0926	0.4837	28	0.0116	0.2031	0.1973	0.0407	2.1901	0.0181	0.0042	True std
						0.0054	0.4688	0.4468	0.0649	1.7756	0.0168	0.0040	Pred std
8	0.0003	0.0286	0.0891	0.4767	50	0.0095	0.2721	0.2048	0.0650	2.4875	0.0207	0.0054	True std
						0.0053	0.4423	0.4700	0.0711	1.7409	0.0148	0.0040	Pred std
9	0.0001	0.0287	0.0970	0.4777	86	0.0136	0.2038	0.1741	0.0362	1.2230	0.0103	0.0033	True std
						0.0076	0.6054	0.4981	0.0943	1.6644	0.0152	0.0049	Pred std
10	0.0003	0.0852	0.0880	0.4884	2124	0.0145	0.1902	0.1736	0.0620	2.3005	0.0160	0.0039	True std
						0.0151	0.3972	0.4183	0.0702	1.9341	0.0149	0.0037	Pred std
11	0.0002	0.0300	0.1025	0.4526	867	0.0139	0.1735	0.1290	0.0661	2.1370	0.0139	0.0034	True std
						0.0084	0.3749	0.4028	0.0728	1.8038	0.0158	0.0040	Pred std
12	0.0004	0.0188	0.2535	0.4945	1283	0.0096	0.0706	0.0779	0.1007	0.9193	0.0069	0.0017	True std
						0.0077	0.3606	0.4202	0.0715	1.8460	0.0165	0.0034	Pred std

13	0.0002	0.0263	0.1016	0.4602	1163	0.0156	0.2172	0.1827	0.0768	1.0687	0.0116	0.0032	True std
						0.0106	0.3961	0.3995	0.0674	1.4860	0.0126	0.0035	Pred std
14	0.0002	0.0301	0.1914	0.4643	1593	0.0129	0.1405	0.1017	0.1683	1.6904	0.0097	0.0018	True std
						0.0196	0.3708	0.4947	0.0822	2.3039	0.0209	0.0038	Pred std
15	0.0002	0.0602	0.2675	0.4573	2530	0.0170	0.2447	0.1774	0.2266	2.1668	0.0123	0.0031	True std
						0.0271	0.4412	0.4737	0.1583	1.9536	0.0167	0.0043	Pred std
16	0.0000	0.0355	0.2773	0.4242	1734	0.0108	0.2053	0.1475	0.3077	1.2440	0.0125	0.0039	True std
						0.0099	0.5685	0.5724	0.3313	1.7762	0.0168	0.0052	Pred std
17	0.0003	0.0365	0.3560	0.4236	2924	0.0082	0.1581	0.0983	0.2216	1.7136	0.0070	0.0014	True std
						0.0152	0.6146	0.6243	0.1856	2.4844	0.0321	0.0059	Pred std
18	0.0002	0.0810	0.1948	0.4194	2162	0.0050	0.7164	0.4255	0.4650	0.9320	0.0192	0.0061	True std
						0.0147	0.7343	0.6980	0.3039	1.7018	0.0192	0.0063	Pred std
19	0.0002	0.0306	0.1764	0.4431	2279	0.0118	0.1911	0.2644	0.1092	1.0977	0.0070	0.0017	True std
						0.0113	0.4807	0.5566	0.1664	2.2130	0.0211	0.0047	Pred std
20	0.0004	0.0571	0.0365	0.3849	13	0.0010	0.0729	0.0721	0.0222	1.2292	0.0091	0.0022	True std
						0.0063	0.3930	0.4305	0.0711	1.5438	0.0170	0.0037	Pred std

Table S5 presents the standard deviation of the measured and predicted values in the test set. The observed experimental uncertainty, expressed as standard deviation from repeated measurements, remains low and stable across most output variables with the exception of the *EC* value, which exhibits comparatively higher variance. However, this deviation is not deemed impactful, as SHAP-based sensitivity analysis (via the PartitionExplainer method) indicates *EC* has minimal influence on the model outputs (discussed later). Additionally, *EC* shows only moderate correlation with cathode temperature, further limiting its significance in the predictive framework. *FE(CO)* also shows higher uncertainty, but this is also the case for the measurements conducted on the different cell assembly and stands true for the entirety of the training dataset.

The model's predictive uncertainty (likely epistemic in nature) is consistently higher (except for a small set of pairs) than the physical measurement's uncertainty. This possibly reflects the model's limited confidence due to the limited number of parameter settings in the training dataset. The higher predictive uncertainty suggests that the model has not fully learned the underlying dynamics for all operational conditions, which can be mitigated in the future with the addition of data of higher diversity and volume, which should help

close the gap between model and measurement uncertainty, improving both reliability and generalizability of the predictions.

Due to the cell-to-cell variability commonly known in the field of CO2RR, it is expected that the model exhibits higher uncertainty on the external test set. **Figure S9** visualizes the uncertainty in the data and in the predictions of the model on the external test set.

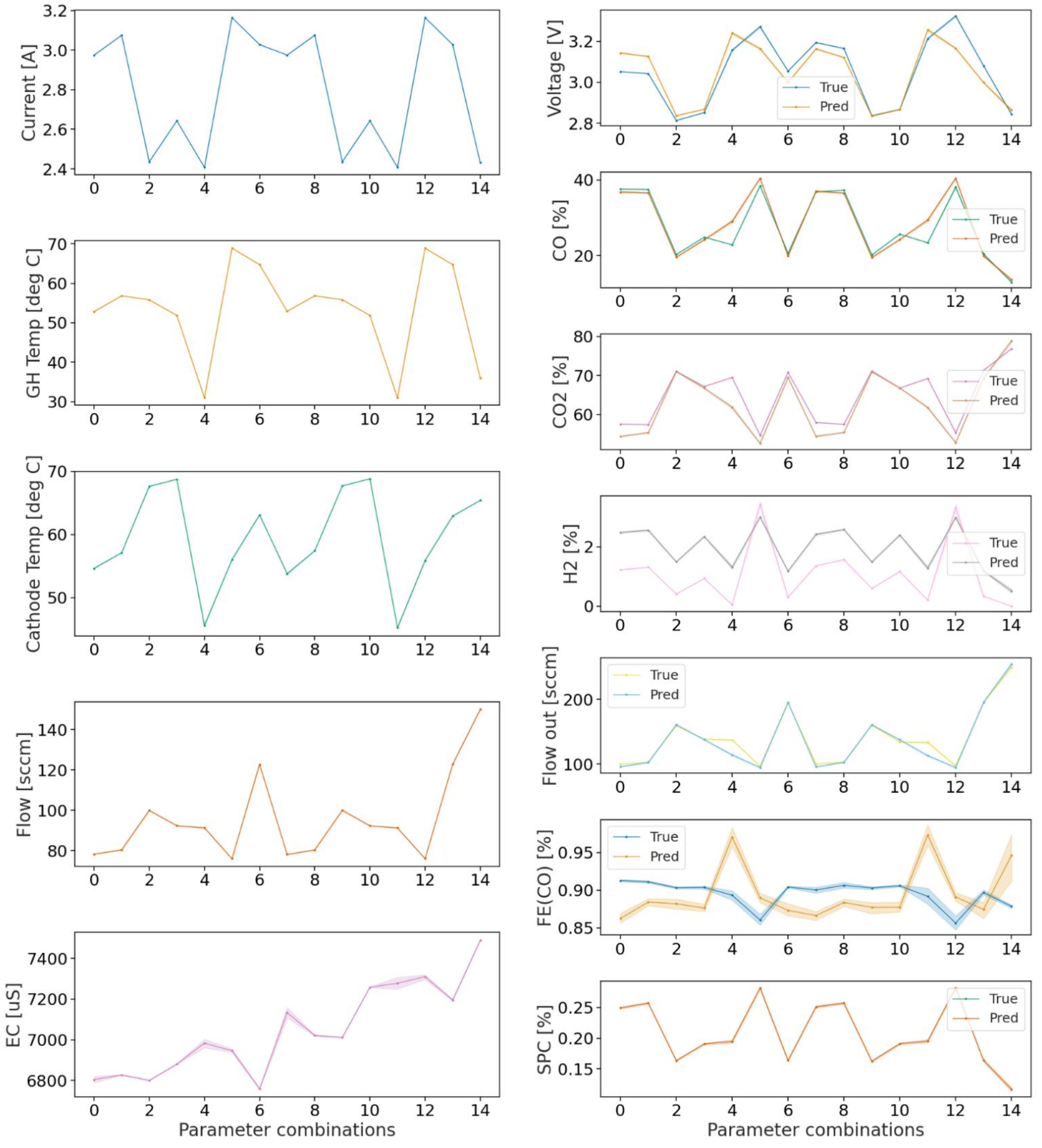

Figure S9. Representing uncertainty in the data and the model predictions on the external test set. The left side shows the mean and the confidence interval for the input values, while on the right side one can observe these values on the ANN predictions, and the true values.

The uncertainty of the measured input parameter settings is comparable to the uncertainty in the case of the test set (**Figure S8**), with the only notable difference being in the value of electric conductivity of the anolyte (*EC*), which has higher variation in the test set. This is later discussed in the section dedicated for model sensitivity analysis, however, it should be noted here also that *EC* does not have high importance in determining either of the output features, and as such, higher variance in the output features should not be attributed to this fact alone.

In the case of the external test set, considerably higher error can be observed on the model side, especially in the case of the predicted H_2 value. Increase in error is to be expected in this case due to the fact that in most cases at least one parameter setting is outside the range that the model was trained on, which in turn can cause the model to respond with a higher variability in the outputs even for small change in the inputs. Also, a high uncertainty in the predicted *FE(CO)* value in the last parameter combination with the following settings is especially conspicuous:

- Cell Temp Setpoint [deg C] = 66
- GH Temp Setpoint [deg C] = 36
- MFC Setpoint [sccm] = 150
- Current Limit Setpoint [A] = 2.432

This can be attributed to the fact that GH and MFC Setpoint both significantly fall outside the range that the model was trained on, which causes more uncertainty in the model predictions even for smaller variations in input data (see **Table S7**).

The prediction performance of the model shows degradation compared to the test set. However, examining the target values *FE(CO) [%]* and *CO2 Conversion Rate [%]*, one can see an overlap in these values. The mean of Faraday Efficiency and conversion rate is predicted moderately well with average distances of 0.0397 and 0.0088 which are ~4 % of the corresponding column means (0.8961 and 0.2084, respectively).

In the case of cell voltage, the average distance (0.0565) is ~2 % of the corresponding column mean (3.0479), which suggests that a varying EC value does not necessarily imply unstable cell operation, even in the case of cell voltage, where *EC* is measured to be the third most important feature in predicting this value (see Model sensitivity analysis), although still with a relatively low feature importance value compared to the first two (Current and Cell temperature).

Table S6. Comparing ANN predicted and true mean values for individual measurement sequences for each parameter setting on the external test set. For more information on table format and colorings refer to **Table S4**.

#	Current [A]	GH Temp [deg C]	Cathode Temp [deg C]	Flow [sccm]	EC [uS]	Voltage [V]	CO [%]	CO2 [%]	H2 [%]	Flow out [sccm]	FE(CO) [%]	CO2 Conversion Rate [%]	Type
0	2.9749	52.8591	54.6361	78.2781	6805	3.0524	37.5572	57.5202	1.2249	99.9987	0.9128	0.2639	True mean
						3.1435	36.8322	54.3997	2.4675	96.2157	0.8626	0.2494	Pred mean
1	3.0750	56.8434	57.1113	80.4380	6828	3.0425	37.4967	57.4182	1.3086	103.3224	0.9110	0.2649	True mean
						3.1259	36.5213	55.3900	2.5603	102.7790	0.8833	0.2569	Pred mean
2	2.4360	55.8340	67.6097	99.9626	6801	2.8135	20.2418	71.0497	0.4095	159.4243	0.9032	0.1674	True mean
						2.8379	19.5218	70.9354	1.4866	160.803	0.8797	0.1631	Pred mean
3	2.6430	51.8734	68.7331	92.3760	6881	2.8522	24.8454	67.1700	0.9333	138.6204	0.9036	0.1967	True mean
						2.8676	24.1809	66.7956	2.3576	137.7224	0.8760	0.1906	Pred mean
4	2.4079	30.9874	45.6015	91.3128	6984	3.1574	22.7946	69.4883	0.0444	137.3984	0.8933	0.1792	True mean
						3.2454	29.2723	61.8959	1.3371	113.8203	0.9745	0.1955	Pred mean
5	3.1640	68.9156	56.0232	76.0108	6947	3.2722	38.4406	54.6427	3.4283	97.0107	0.8604	0.2724	True mean
						3.1651	40.3931	52.5928	2.9997	94.7303	0.8870	0.2808	Pred mean
6	3.0278	64.7573	63.0959	122.8108	6760	3.0550	20.6106	70.7927	0.2995	194.6499	0.9042	0.1696	True mean
						2.9998	19.8643	69.4286	1.1804	195.5750	0.8759	0.1643	Pred mean
7	2.9749	52.9100	53.7922	78.1145	7134	3.1946	36.8621	57.9676	1.3606	100.6624	0.9002	0.2608	True mean
						3.1634	37.0125	54.2282	2.4073	95.5471	0.8611	0.2495	Pred mean
8	3.0750	56.8517	57.4215	80.3912	7022	3.1655	37.3014	57.4729	1.5631	103.3195	0.9064	0.2637	True mean
						3.1220	36.632	55.3127	2.5949	102.5721	0.8847	0.2574	Pred mean
9	2.4360	55.8401	67.6939	100.0298	7012	2.8374	20.129	71.1607	0.5926	160.2209	0.9029	0.1673	True mean
						2.8350	19.5554	71.0372	1.4942	161.0644	0.8827	0.1635	Pred mean
10	2.6430	51.8763	68.8062	92.3286	7258	2.8678	25.6298	66.7696	1.1651	134.2476	0.9058	0.1972	True mean
						2.8668	24.1914	66.8133	2.4021	137.586	0.8756	0.1907	Pred mean
11	2.4080	30.9796	45.2475	91.2795	7278	3.2140	23.3506	69.2196	0.2008	133.571	0.8917	0.1789	True mean
						3.2568	29.5225	61.9983	1.3209	113.7519	0.9829	0.1972	Pred mean

#	Current [A]	GH Temp [deg C]	Cathode Temp [deg C]	Flow [sccm]	EC [uS]	Voltage [V]	CO [%]	CO2 [%]	H2 [%]	Flow out [sccm]	FE(CO) [%]	CO2 Conversion Rate [%]	Type
12	3.1640	68.9121	55.8894	76.0218	7310	3.3244	38.0963	55.3402	3.3175	97.5518	0.8563	0.2711	True mean
						3.1655	40.4068	52.7455	2.9745	95.0483	0.8902	0.2818	Pred mean
13	3.0278	64.7607	62.8956	122.8685	7195	3.0811	20.4101	71.3092	0.3253	195.1047	0.8969	0.1681	True mean
						3.0002	19.8120	69.3938	1.1780	195.3055	0.8723	0.1635	Pred mean
14	2.4310	35.9660	65.3864	150.0379	7488	2.8438	12.8816	76.8495	0.0000	249.5994	0.8784	0.1083	True mean
						2.8682	13.7557	78.8951	0.4853	255.7825	0.9662	0.1191	Pred mean
					**Column mean**	3.0479	28.1374	63.8678	1.5140	138.7669	0.8961	0.2084	
					**Averaged distance of values**	0.0565	1.6508	2.4362	0.9715	4.6606	0.0397	0.0088	

In **Table S6** it can be seen that the model can struggle to predict parameter settings where two or more parameters fall outside the range of the training data. For example, the value setting for the 14th index produces high uncertainty in the model (as shown on **Figure S9**), with a mean value also being off by a higher margin as opposed to other predictions, where the GH temperature and the Flow rate is out of bounds. Similar effects can be observed when both temperature values fall outside the specified range for the 4th and 11th indexed rows, as here the predicted output flow rate is ~15% less than the true output flow, which results in a higher Faraday Efficiency prediction. At the 0th and 1st indexed rows we can see that the model performs significantly better, even though the cell temperature falls below 60 ˚C (the minimum cell temperature in the training data). This can be observed in setting 3 and 5 too, where inputs are relatively close to ranges of the training data. This demonstrates the model's ability to generalize to some extent even outside the training data range. However, it shows its limitations too, i.e. when the model is used well beyond the training data ranges, it can produce unrealistic results.

Table S7. Comparing ANN predicted and true std values for individual measurement sequences for each parameter setting on the external test set.

#	Current [A]	GH Temp [deg C]	Cathode Temp [deg C]	Flow [sccm]	EC [uS]	Voltage [V]	CO [%]	CO2 [%]	H2 [%]	Flow out [sccm]	FE(CO) [%]	CO2 Conversion Rate [%]	Type

0	0.0003	0.0322	0.1759	0.4244	52	0.0009	0.0617	0.0665	0.0173	0.4732	0.0048	0.0020	True std
						0.0091	0.6501	0.6542	0.1069	1.9128	0.0191	0.0054	Pred std
1	0.0001	0.0352	0.0327	0.4541	7	0.0005	0.0749	0.0849	0.0158	0.4181	0.0046	0.002	True std
						0.0064	0.5199	0.4818	0.0766	1.503	0.0143	0.0044	Pred std
2	0.0001	0.0334	0.0495	0.4990	6	0.0005	0.0479	0.0696	0.0149	0.6288	0.0043	0.0011	True std
						0.0067	0.4023	0.5193	0.0633	2.2711	0.0221	0.0042	Pred std
3	0.0002	0.0205	0.0444	0.4646	8	0.0010	0.0919	0.1025	0.0137	0.8864	0.0061	0.0016	True std
						0.0049	0.3779	0.5045	0.0669	1.9836	0.0202	0.0043	Pred std
4	0.0003	0.0245	0.3618	0.4489	58	0.0108	0.1759	0.1449	0.0165	2.4406	0.0167	0.0035	True std
						0.0199	1.038	1.1355	0.1949	3.4511	0.0415	0.0085	Pred std
5	0.0002	0.0335	0.1201	0.3982	29	0.0105	0.6885	0.3153	0.3509	1.5382	0.0221	0.0072	True std
						0.0097	0.6921	0.5881	0.1106	2.0319	0.0215	0.007	Pred std
6	0.0004	0.0198	0.0333	0.5913	11	0.0009	0.0753	0.0861	0.0111	0.7739	0.0045	0.0011	True std
						0.0095	0.5254	0.5212	0.0931	2.694	0.0237	0.0045	Pred std
7	0.0002	0.0302	0.2718	0.387	86	0.0036	0.1987	0.1370	0.0187	1.322	0.0119	0.0037	True std
						0.0105	0.6108	0.5614	0.1020	1.7756	0.0183	0.0052	Pred std
8	0.0002	0.0345	0.0289	0.4204	10	0.0014	0.0953	0.0913	0.0243	1.247	0.0113	0.0037	True std
						0.0061	0.4699	0.4318	0.0820	1.4458	0.0146	0.0045	Pred std
9	0.0000	0.0348	0.0281	0.5035	8	0.0008	0.066	0.0691	0.0091	1.0800	0.0062	0.0013	True std
						0.0061	0.3575	0.4546	0.0662	2.0721	0.0188	0.0035	Pred std
10	0.0002	0.0157	0.0326	0.4942	7	0.0009	0.0577	0.0871	0.0137	0.6400	0.0047	0.0015	True std
						0.0047	0.3950	0.4513	0.0767	1.8108	0.0185	0.0042	Pred std
11	0.0000	0.0286	0.3554	0.4768	90	0.0068	0.2181	0.1822	0.0201	4.2099	0.0311	0.0063	True std
						0.0196	1.0621	0.9466	0.1921	3.8217	0.0435	0.0088	Pred std
12	0.0002	0.0344	0.1049	0.3917	37	0.0114	0.9477	0.4185	0.4276	1.7241	0.0282	0.0092	True std
						0.0103	0.7184	0.6648	0.1116	1.7780	0.0187	0.0061	Pred std
13	0.0004	0.0231	0.0315	0.5465	13	0.0028	0.1593	0.1522	0.015	1.3048	0.0092	0.0019	True std
						0.009	0.4897	0.5877	0.0875	3.1620	0.0270	0.005	Pred std
14	0.0002	0.0299	0.0336	0.3126	8	0.0008	0.071	0.0768	0.0000	1.3996	0.0071	0.0009	True std
						0.0197	1.2631	1.3293	0.2443	5.2965	0.0954	0.0117	Pred std

From **Table S7** we can see that the model usually has more uncertainty (e.g., parameter setting #14) than the real system itself. This is still the case even when the predicted mean is close to the true value. This is a limitation of the current model, which later could be mitigated by including additional high quality data points in the training set.

Analysis of Possible Feature Correlations

It is known that not all model sensitivity analysis tools can be used safely in the presence of stronger input correlations. To mitigate this issue, we analyzed the possible correlations between input data (**Figure S10**) to ensure our chosen method's validity.

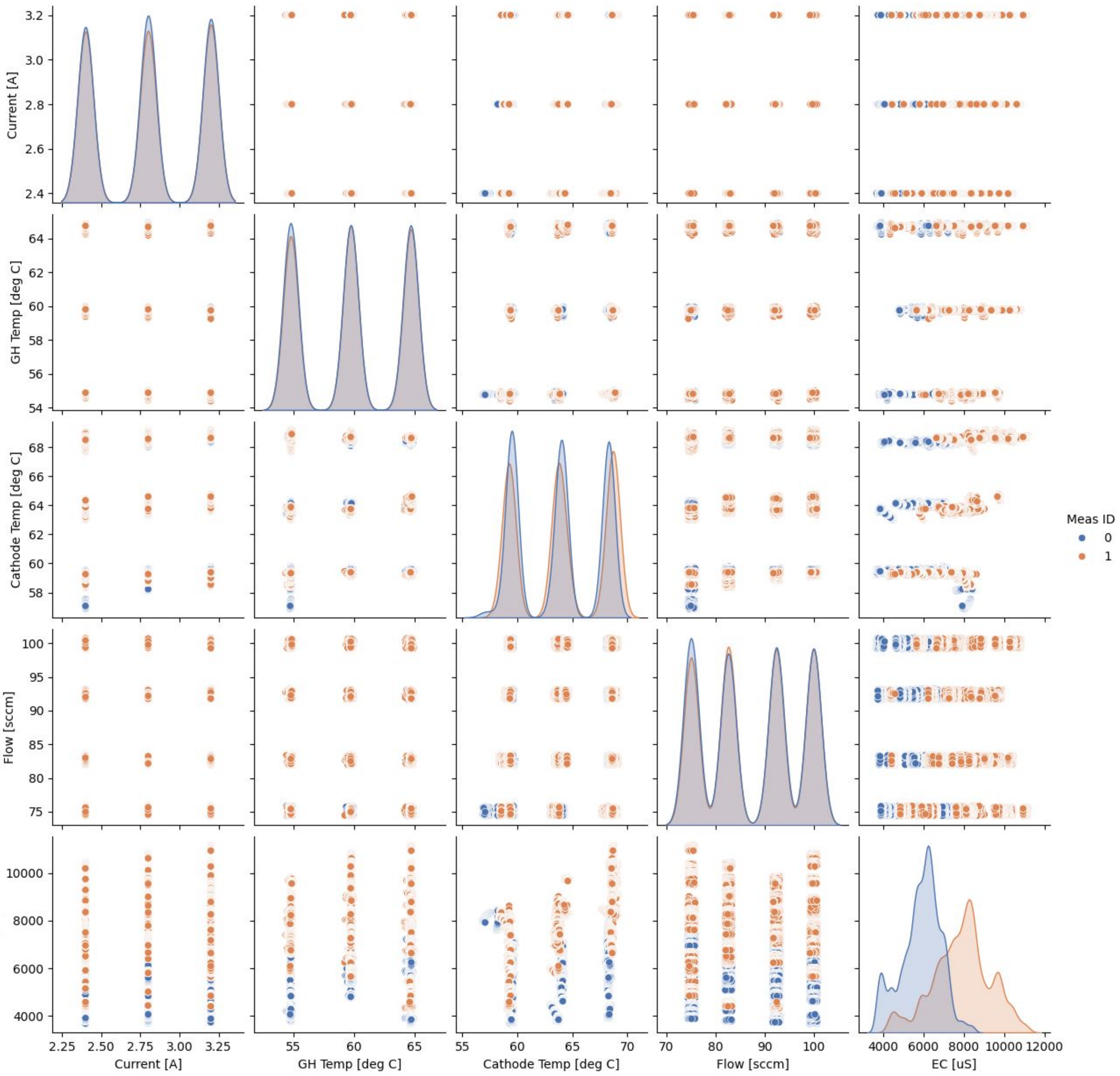

Figure S10. Correlation between different input parameters in the model development dataset. Meas ID signifies the repeated measurement sequences used for model development, with the 0th ID corresponding to

the first, and the 1st ID corresponding to the repeated measurements. The diagonal in the plot shows the distribution of the different input feature values, while the scatter plots show the individual measurement points sample-by-sample. These scatter plots help visualize obvious patterns of correlations between the input features, if any.

The distribution plots in **Figure S10** show the different control variable settings, as the majority of samples gather around the specific settings and are easily separated by the different maximums. These local maximums correspond to the settings in **Table 1** in the m-s. There are no obvious correlations between any of the input features based on the plot, however, a moderate positive correlation between *EC* and *Cathode Temp* could be observed, implying the temperature dependence of the measured conductivity values (despite using a temperature-compensated conductivity probe). To quantify this correlation, we calculated the Pearson correlation coefficient (PCC) for every pair of input features.[3]

Table S8. Correlation between input features, numerically representing **Figure S10**.

	Current [A]	GH Temp [deg C]	Cathode Temp [deg C]	Flow [sccm]	EC [uS]
**Current [A]**	1.0000	-0.0147	0.0371	-0.0131	0.0309
**GH Temp [deg C]**	-0.0147	1.0000	0.0298	-0.0036	-0.0222
**Cathode Temp [deg C]**	0.0371	0.0298	1.0000	0.0237	0.3744
**Flow [sccm]**	-0.0131	-0.0036	0.0237	1.0000	-0.0368
**EC [uS]**	0.0309	-0.0222	0.3744	-0.0368	1.0000

Table S8 shows that there is no significant correlation between most of the input features, though a moderate correlation between *Cathode Temp* and *EC* can be observed. Note that, although *EC* appears as a model input, it is not a controllable parameter (as mentioned in the m-s).

Model Sensitivity Analysis

In traditional sensitivity analysis (SA) methods such as one-at-a-time (OAT), only one model factor is changed at a time while others are kept fixed. In each iteration, the algorithm selects an independent (in our case input) variable to perturb so it can analyze how that particular variable affects the model outcome. This method is model agnostic; however, one significant drawback is that it cannot detect feature interactions or higher order, nonlinear effects. Another option is using Sobol' indices; however, classical Sobol' sensitivity indices are defined under the assumption that the input variables are independent.[4] For models featuring dependent inputs, this may lead to a wrong interpretation because the sensitivity induced by the dependence between two factors is implicitly included in their Sobol indices.[5]

A different approach is to use Shapley effects instead of Sobol' indices.[6] The Shapley value concept from cooperative game theory allocates the total output variance (the "value") among inputs fairly, accounting for correlations and interactions. Shapley indices have the advantages of always summing to one and being well-defined under dependence. Iooss *et al.* show that Shapley effects allocate the mutual contribution (due to correlation and interaction) of a group of inputs to each individual input, effectively disentangling shared effects.[7] Shapley-based measures also give more interpretable attributions when inputs are dependent.[7,8] In practice, many modern sensitivity-analysis toolkits (e.g. SHAP) now offer Shapley-effect estimation as the recommended approach for correlated inputs when using typical machine learning models.[9]

When applying Shapley values to machine learning, it can be interpreted by making the following equivalences.

- **Features = Players**: Input features are considered to be players of a cooperative game.
- **Model Prediction = Payout**: The model's prediction could be thought of as the payout.
- **Baseline/Reference Value**: SHAP often uses the average model prediction (over the training data, or an optionally aggregated (e.g., with K-*means*) subset of it) as a baseline. SHAP values then explain

how each feature shifts the prediction from this baseline expectation to the specific instance's predicted value.

Eventually, we chose SHAP for its proven ability to explain even black box models, such as Deep Neural Networks (DNNs). SHAP [10] also has a proven record of being consistent and accurate [11,12] even in the presence of feature interactions. Furthermore, for small number of input features (such as the case in this work), it could also be argued that it is a more adequate choice over other methods, such as constructing Sobol' indices, due to its compelling properties.[13–15]

With the use of SHAP values we were able to rank each input feature based on its importance score for a specific output feature. In the following sections, we will discuss how different control parameters might affect the experiment based on the extracted Shapley values.

In selecting a specific SHAP method for approximating Shapley values, we considered the fact that the input features (*Current*, *GH Temp*, *Cell Temp*, and *Flow*) except for the non-controllable *EC* parameter are largely independent, with no strong evidence of direct interactions, with *EC* and *Cathode Temp* being moderately dependent on each other. Some implementations (e.g., KernelSHAP) make the assumption of feature independence, and as such, we only considered methods that account for possible feature correlations.[9]

The Partition SHAP method provides a model-agnostic approach for explaining model outputs by computing Shapley values through a hierarchical feature clustering, yielding the Owen values from cooperative game theory.[16] Unlike Kernel or Sampling-based SHAP methods, which scale exponentially with the number of features, Partition SHAP achieves exact computation with quadratic complexity when employing a balanced partition tree. A notable advantage of this approach is its capacity to account for feature dependencies. When correlated features are grouped within the hierarchy, Partition SHAP allocates their collective contribution without requiring perturbations that disrupt their statistical relationships.

SHAP Method for Sensitivity Analysis

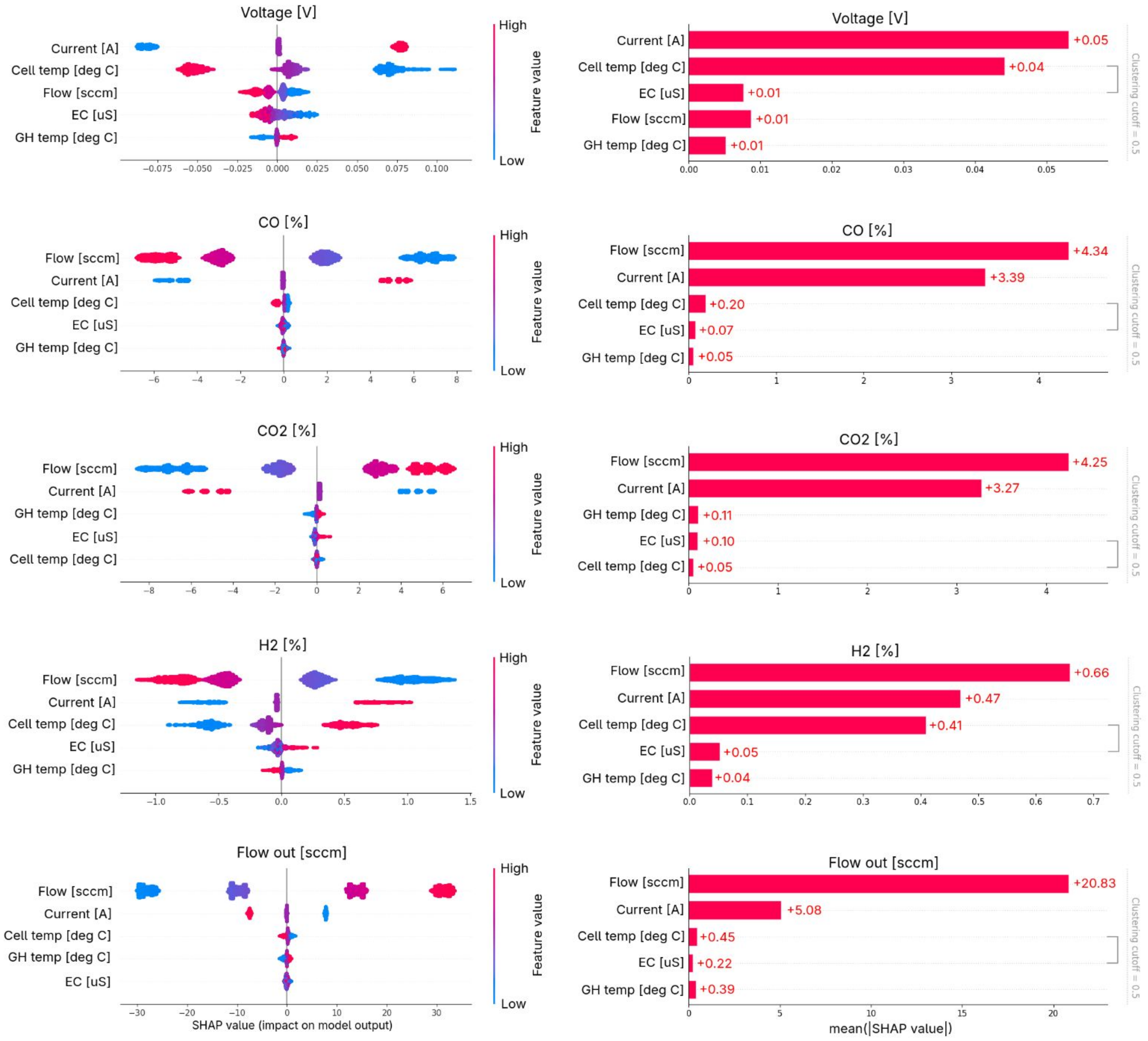

Figure S11. On the left side we can see the SHAP based ANN model sensitivity where all the input parameters are sorted based on their impact on model output which can be observed on the right colorbar. On the right, SHAP values are aggregated over individual input parameters (taking the mean), so that they show their global feature importance, and also show the groupings that the Partition SHAP method accounted for, which may cause some reordering compared to the local contributions, as Partition SHAP groups correlating features together.

Examining **Figure S11** one can immediately see how each input feature contributes to different model outcomes. On the first row we can see that cell voltage is most affected by the applied current, and the temperature of the cell. Positively correlating in the case of electric current while negatively correlating with the temperature. The grouping in values (on the left figure) can be attributed to the fact that the total 108 parameter combinations were formed of 4 flow rates, and 3 different levels for the other 3 parameters ($3^3 \times 4$ combinations). The ordering of the features, however, depends on the mean attribution of one particular input feature. For example, this means that the cell voltage on the first row is mostly affected by the applied current. The concentration of gaseous elements mostly depends on the flow rate and are highly affected by the current. This is caused by both the different dilution and the selectivity change at different flow rates together. The H_2 concentration in the gas stream increases with the cell temperature, most probably due to the larger amount of water crossing the membrane from the anode to the cathode, hence promoting H_2 formation. Lastly, the output flow rate is affected mostly by the inlet flow rate, with a positive correlation, while also negatively affected by the set current. This latter is caused by the loss of carbon dioxide due to its reaction with the electrochemically generated hydroxide ions ($CO_2 + 2e^- + H_2O \rightarrow CO + 2\mathrm{OH}^-$ & $CO_2 + 2OH^- \rightarrow CO_3^{2-} + H_2O$), and the transport of the formed carbonate ions to the anode.

Overall, the SHAP analysis provided clear and intuitive insights into some of the input features' influence on model predictions. The strong effects of current and cell temperature reflect their effect in driving the electrochemical processes, which matches well with our current understanding. The grouped values showed correlation between some of the input features, namely EC and Cathode Temp, which is validated by our previous findings when analyzing for possible input correlations. These values in turn play a pivotial role in determining the cell voltage. These findings demonstrate that SHAP is not only effective for model interpretation but also valuable for exploring how key parameters shape system behavior.

References

(1) Akiba, T.; Sano, S.; Yanase, T.; Ohta, T.; Koyama, M. Optuna: A Next-Generation Hyperparameter Optimization Framework. *Proceedings of the 25th ACM SIGKDD international conference on knowledge discovery & data mining* **2019**, 2623–2631.

(2) Deb, K.; Pratap, A.; Agarwal, S.; Meyarivan, T. A Fast and Elitist Multiobjective Genetic Algorithm: NSGA-II. *IEEE Transactions on Evolutionary Computation* **2002**, *6* (2), 182–197. https://doi.org/10.1109/4235.996017.

(3) Benesty, J.; Chen, J.; Huang, Y.; Cohen, I. Pearson Correlation Coefficient; **2009**; 1–4. https://doi.org/10.1007/978-3-642-00296-0_5.

(4) Sobol, I. M. *Global Sensitivity Indices for Nonlinear Mathematical Models and Their Monte Carlo Estimates*; **2001**, *55* (1-3), 271-280. https://doi.org/10.1016/S0378-4754(00)00270-6.

(5) Chastaing, G.; Gamboa, F.; Prieur, C. Generalized Sobol Sensitivity Indices for Dependent Variables: Numerical Methods. *J Stat Comput Simul* **2015**, *85* (7), 1306–1333. https://doi.org/10.1080/00949655.2014.960415.

(6) Song, E.; Nelson, B. L.; Staum, J. Shapley Effects for Global Sensitivity Analysis: Theory and Computation. *SIAM/ASA Journal on Uncertainty Quantification* **2016**, *4* (1), 1060–1083. https://doi.org/10.1137/15M1048070.

(7) Iooss, B.; Prieur, C. SHAPLEY EFFECTS FOR SENSITIVITY ANALYSIS WITH CORRELATED INPUTS: COMPARISONS WITH SOBOL' INDICES, NUMERICAL ESTIMATION AND APPLICATIONS. *Int J Uncertain Quantif* **2019**, *9* (5), 493–514. https://doi.org/10.1615/Int.J.UncertaintyQuantification.2019028372.

(8) Benoumechiara, N.; Elie-Dit-Cosaque, K. Shapley Effects for Sensitivity Analysis with Dependent Inputs: Bootstrap and Kriging-Based Algorithms. *ESAIM Proc Surv* **2019**, *65*, 266–293. https://doi.org/10.1051/proc/201965266.

(9) Lundberg, S. M.; Allen, P. G.; Lee, S.-I. A Unified Approach to Interpreting Model Predictions. *Proceedings of the 31st International Conference on Neural Information Processing Systems* **2017**, 4768–4777. https://doi.org/10.5555/3295222.3295230.

(10) Yang, C.; Guan, X.; Xu, Q.; Xing, W.; Chen, X.; Chen, J.; Jia, P. How Can SHAP (SHapley Additive ExPlanations) Interpretations Improve Deep Learning Based Urban Cellular Automata Model? *Comput Environ Urban Syst* **2024**, *111*, 102133. https://doi.org/10.1016/j.compenvurbsys.2024.102133.

(11) Nohara, Y.; Matsumoto, K.; Soejima, H.; Nakashima, N. Explanation of Machine Learning Models Using Shapley Additive Explanation and Application for Real Data in Hospital. *Comput Methods Programs Biomed* **2022**, *214*, 106584. https://doi.org/10.1016/j.cmpb.2021.106584.

(12) El Bilali, A.; Abdeslam, T.; Ayoub, N.; Lamane, H.; Ezzaouini, M. A.; Elbeltagi, A. An Interpretable Machine Learning Approach Based on DNN, SVR, Extra Tree, and XGBoost Models

for Predicting Daily Pan Evaporation. *J Environ Manage* **2023**, *327*, 116890. https://doi.org/10.1016/j.jenvman.2022.116890.

(13) Owen, A. B. Sobol' Indices and Shapley Value. *SIAM/ASA Journal on Uncertainty Quantification* **2014**, *2* (1), 245–251. https://doi.org/10.1137/130936233.

(14) Vuillod, B.; Montemurro, M.; Panettieri, E.; Hallo, L. A Comparison between Sobol's Indices and Shapley's Effect for Global Sensitivity Analysis of Systems with Independent Input Variables. *Reliab Eng Syst Saf* **2023**, *234*, 109177. https://doi.org/10.1016/j.ress.2023.109177.

(15) Plischke, E.; Rabitti, G.; Borgonovo, E. Computing Shapley Effects for Sensitivity Analysis. *SIAM/ASA Journal on Uncertainty Quantification* **2021**, *9* (4), 1411–1437. https://doi.org/10.1137/19M1304738.

(16) Owen, G. Values of Games with a Priori Unions; **1977**, 76–88. https://doi.org/10.1007/978-3-642-45494-3_7.
